# *In Vitro* and *In Vivo* Characterization of the Alkaloid Nuciferine

**DOI:** 10.1371/journal.pone.0150602

**Published:** 2016-03-10

**Authors:** Martilias S. Farrell, John D. McCorvy, Xi-Ping Huang, Daniel J. Urban, Kate L. White, Patrick M. Giguere, Allison K. Doak, Alison I. Bernstein, Kristen A. Stout, Su Mi Park, Ramona M. Rodriguiz, Bradley W. Gray, William S. Hyatt, Andrew P. Norwood, Kevin A. Webster, Brenda M. Gannon, Gary W. Miller, Joseph H. Porter, Brian K. Shoichet, William E. Fantegrossi, William C. Wetsel, Bryan L. Roth

**Affiliations:** 1 Department of Pharmacology, University of North Carolina School of Medicine, Chapel Hill, North Carolina, United States of America; 2 Department of Psychiatry, University of North Carolina School of Medicine, Chapel Hill, North Carolina, United States of America; 3 Program in Neuroscience, University of North Carolina School of Medicine, Chapel Hill, North Carolina, United States of America; 4 Lineberger Comprehensive Cancer Center, University of North Carolina School of Medicine, Chapel Hill, North Carolina, United States of America; 5 Carolina Institute for Developmental Disabilities, University of North Carolina School of Medicine, Chapel Hill, North Carolina, United States of America; 6 Division of Chemical Biology and Medicinal Chemistry, School of Pharmacy, University of North Carolina School of Medicine, Chapel Hill, North Carolina, United States of America; 7 National Institute of Mental Health Psychoactive Drug Screening Program, University of North Carolina School of Medicine, Chapel Hill, North Carolina, United States of America; 8 Department of Pharmacology and Toxicology, University of Arkansas for Medical Sciences, Little Rock, Arkansas, United States of America; 9 Department of Psychology, Virginia Commonwealth University, Richmond, Virginia, United States of America; 10 Department of Pharmaceutical Chemistry, University of California San Francisco, San Francisco, California, United States of America; 11 Department of Environmental Health, Rollins School of Public Health and Center for Neurodegenerative Diseases, Emory University, Atlanta, Georgia, United States of America; 12 Departments of Psychiatry and Behavioral Sciences, Cell Biology, and Neurobiology, Mouse Behavioral and Neuroendocrine Analysis Core Facility, Duke University Medical Center, Durham, North Carolina, United States of America; Macau University of Science and Technology, MACAO

## Abstract

**Rationale:**

The sacred lotus (*Nelumbo nucifera*) contains many phytochemicals and has a history of human use. To determine which compounds may be responsible for reported psychotropic effects, we used *in silico* predictions of the identified phytochemicals. Nuciferine, an alkaloid component of *Nelumbo nucifera* and *Nymphaea caerulea*, had a predicted molecular profile similar to antipsychotic compounds. Our study characterizes nuciferine using *in vitro* and *in vivo* pharmacological assays.

**Methods:**

Nuciferine was first characterized *in silico* using the similarity ensemble approach, and was followed by further characterization and validation using the Psychoactive Drug Screening Program of the National Institute of Mental Health. Nuciferine was then tested *in vivo* in the head-twitch response, pre-pulse inhibition, hyperlocomotor activity, and drug discrimination paradigms.

**Results:**

Nuciferine shares a receptor profile similar to aripiprazole-like antipsychotic drugs. Nuciferine was an antagonist at 5-HT_2A_, 5-HT_2C_, and 5-HT_2B_, an inverse agonist at 5-HT_7_, a partial agonist at D_2,_ D_5_ and 5-HT_6_, an agonist at 5-HT_1A_ and D_4_ receptors, and inhibited the dopamine transporter. In rodent models relevant to antipsychotic drug action, nuciferine blocked head-twitch responses and discriminative stimulus effects of a 5-HT_2A_ agonist, substituted for clozapine discriminative stimulus, enhanced amphetamine induced locomotor activity, inhibited phencyclidine (PCP)-induced locomotor activity, and rescued PCP-induced disruption of prepulse inhibition without induction of catalepsy.

**Conclusions:**

The molecular profile of nuciferine was similar but not identical to that shared with several approved antipsychotic drugs suggesting that nuciferine has atypical antipsychotic-like actions.

## 1. Introduction

**Fig 1:**
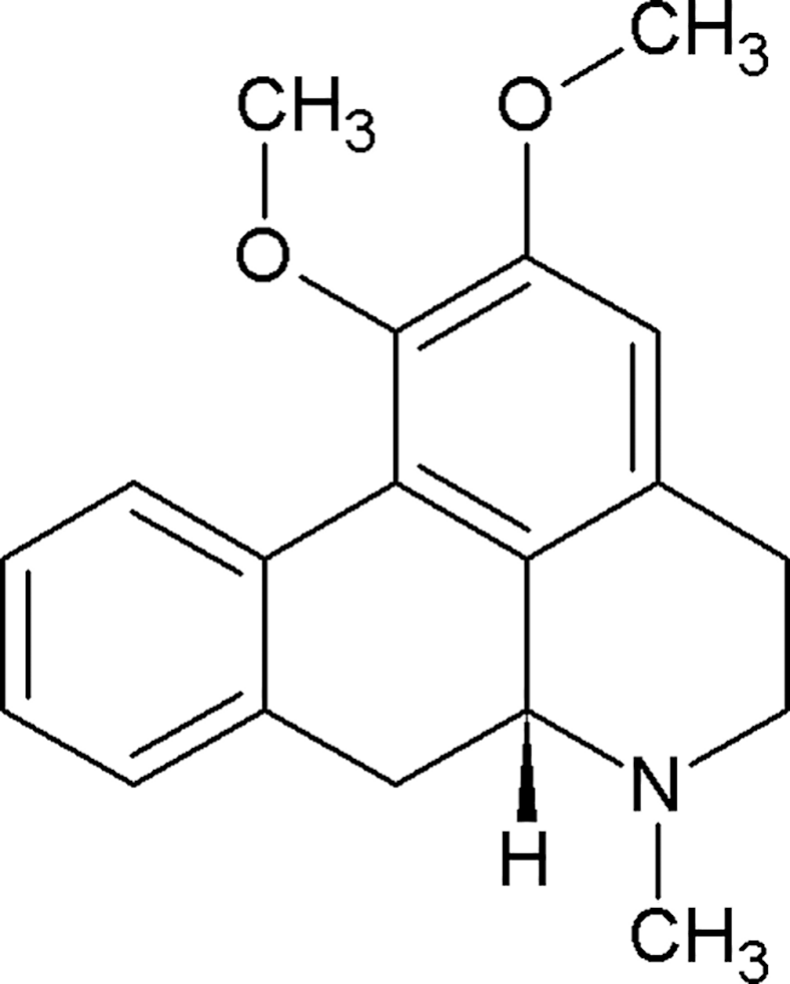
The chemical structure of nuciferine.

The lotus plants, *Nelumbo nucifera* and *Nymphaue caerulea*, have been used by cultures, both past and present, for their medicinal properties.[1] In eastern medicine, one of the cited potential medical effects of the lotus is “calming emotional disturbance”.[2] The alkaloid nuciferine (Fig 1) is thought to be responsible for the psychotropic effects of *Nelumbo nucifera* and *Nymphaea caerulea*, though its pharmacological properties are not entirely clear. Macko and colleagues [3] observed that nuciferine produces effects similar to those of the antipsychotic chlorpromazine in rodents. Bhattacharya and colleagues [4] observed that nuciferine produces antipsychotic-like behavior in rats, including inhibition of the conditioned avoidance response and amphetamine-induced behaviors. The behavioral effects previously observed in rodents, in addition to the cited potential medical effects of the lotus in eastern medicine, led the authors to hypothesize that nuciferine has a pharmacological profile similar to that of antipsychotic medications.

**Fig 2:**
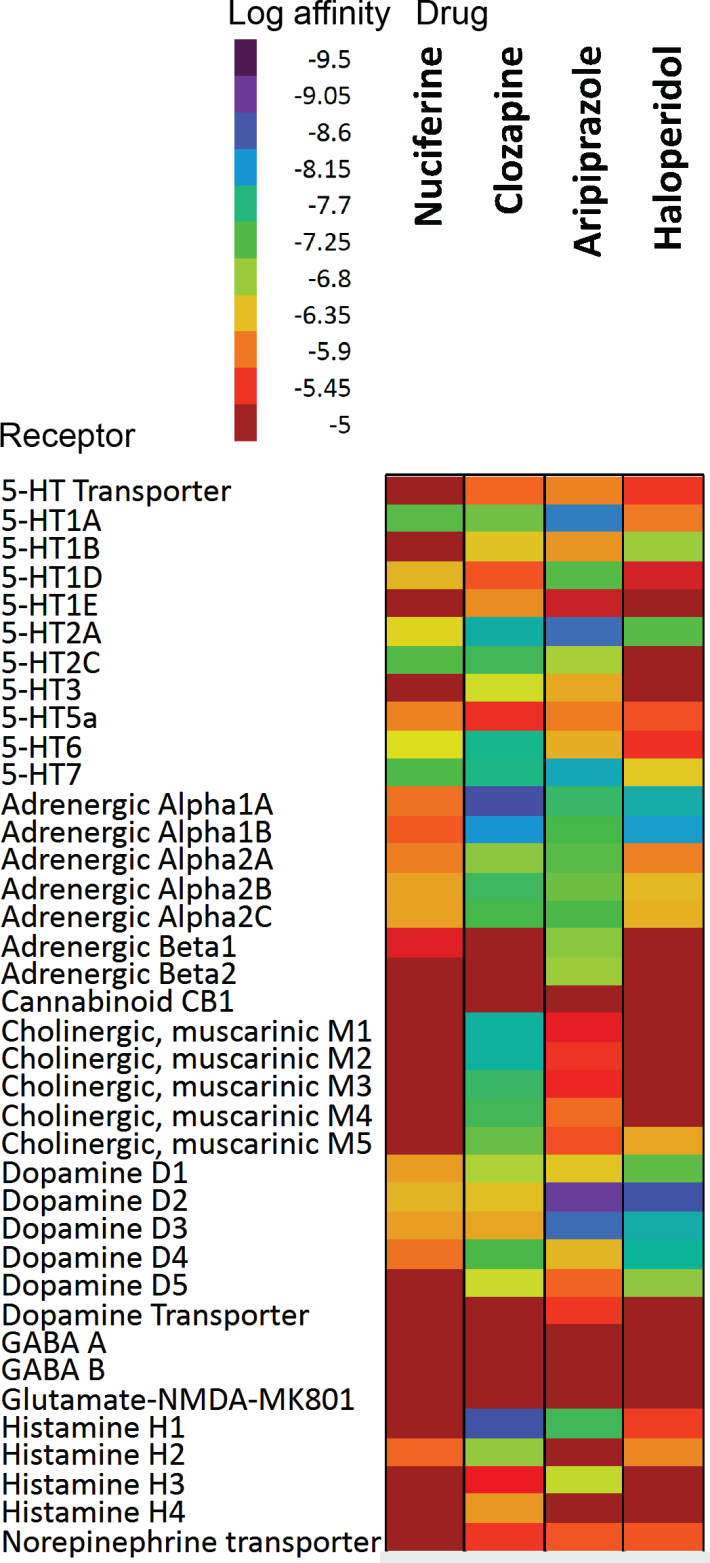
Comparison of the polypharmacology of nuciferine with atypical and typical antipsychotics. The empirical affinity values of three antipsychotics are shown (clozapine, haloperidol, aripiprazole). The empirical nuciferine affinity profile from this study is shown in comparison. Values for clozapine, haloperidol, and aripiprazole compiled from data available on the PDSP website accessed 20150428. Only “PDSP verified” data was used for the figure. Data entries of “>10,000” were entered as 10,000 μM.

Most antipsychotics share common G protein-coupled receptor (GPCR) targets including the D_2_ dopamine and 5-HT_2A_ serotonin receptors. [5–7] Beyond these shared targets, antipsychotic compounds exhibit diverse receptor affinity profiles. For example, the atypical antipsychotic clozapine binds with nanomolar affinities to nearly 50 targets (Fig 2). [8] This polypharmacological profile has been suggested as a path forward in therapeutic drug development.[6, 9] Patterns of pharmacological activity responsible for antipsychotic efficacy remain the subject of ongoing investigations. This polypharmacology approach presents a conundrum for drug discovery efforts as it is impossible to design a compound for a polypharmacological “target” (a pattern of molecular activity that engenders therapeutic efficacy) that has not yet been elucidated. We therefore used ethnobotanical records of *Nelumbo nucifera* and *Nymphaea caerulea* medicinal properties as suggestive evidence for potential therapeutic efficacy of a novel polypharmacological profile. Our *in silico* predictions of all phytochemicals identified in *Nelumbo nucifera* suggest that nuciferine (and its metabolites) cross the blood-brain barrier and have multiple protein targets. Furthermore, nuciferine has been shown to cross the blood brain barrier in rats.[10] These predictions and previously reported data suggest that nuciferine has a rich polypharmacology that is responsible for its psychotropic effects. We therefore investigated the *in vitro* and *in vivo* properties of nuciferine using cell-based pharmacology assays and animal behavioral models of antipsychotic drug action.

## 2. Materials and Methods

### 2.1 Drugs

Nuciferine was purchased from Sequoia Research Products (Pangbourne, United Kingdom) and Angene (Hong Kong, China) and was dissolved in DMSO at 10 mM for in vitro studies or in 0.9% saline with 1 drop 85% lactic acid per 50 ml for animal studies. D-amphetamine (AMPH), phencyclidine (PCP), and 2,5-dimethoxy-4-iodoamphetamine (DOI) were purchased from Sigma-Aldrich (St. Louis, MO, USA) and were dissolved in 0.9% saline. Clozapine (Sigma-Aldrich) was dissolved in 0.2% acetic acid—2% cyclodextran solution. For animal studies, all drugs were administered intraperitoneally unless otherwise noted.

### 2.2 Bioinformatics

The profile of phytochemicals in *Nelumbo nucifera* was obtained from Mukherjee et al.[11] The similarity ensemble approach (SEA) was utilized to predict molecular targets for each phytochemical, using Scitegic ECFP4 fingerprints on a target panel extracted from a “binding” subset of ChEMBL-12 [12] and standardized as previously described. [13, 14] Briefly, The SEA [13–15] uses the chemical similarity of a bait molecule, against that of a set of ligands annotated to a target, to predict whether the bait molecule will modulate that target. Briefly, SEA calculates the similarity of the bait molecule to every annotated ligand, typically using topological fingerprints such as ECFP4. Similarity is calculated as the Tanimoto coefficient (Tc), the number of feature (bits) in common between the bait molecule and any given ligand, divided by the total number of features (bits) in the two molecules; identical molecules will have Tc values of 1.0. The Tc values above a threshold value against all the ligands for the target are averaged, and that Tc is compared to that expected for a set of ligands of similar size that would be expected at random. An E-value is calculated by calculating a Z-score for the observed average Tc vs. the ligand set, then plotting this value against an extreme value distribution and using the BLAST sequence comparison machinery. This E-value represents the likelihood of seeing the similarity one does, between the bait molecule and the known ligands for any given target, compared to what one would expect at random. This calculation is repeated against all of the > 2500 targets in ChEMBL [16] (https://www.ebi.ac.uk/chembl/). The blood-brain barrier penetrability of each compound was predicted using the online blood-brain barrier prediction (BBB) server [17] (http://www.cbligand.org/BBB/).

### 2.3 Psychoactive drug screening program affinity and functional profiling

The NIMH Psychoactive Drug Screening Program (PDSP) has published standardized methods for radioligand binding assays and functional assays. [9, 18–20] Full details of the methods used in the radioligand receptor assays and the functional assays are described in the PDSP Assay Protocol book (http://pdsp.med.unc.edu/pdspw/binding.php).

For affinity determination, nuciferine was subjected to primary radioligand binding assays tested at a single 10 μM concentration to displace 50% of the radioligand at a given receptor target. If a more than 50% of the radioligand was displaced, nuciferine was selected for a secondary binding assay tested at 11 concentrations in triplicate in competition with the radioligand to generate an IC50 and Ki. Binding assays were performed in 96-well plates with 125 μL per well in appropriate binding buffer using radioligand at or near the Kd. Plates are incubated at room temperature in the dark for 90 min. Reactions are stopped by vacuum filtrations onto 0.3% polyethyleneimine soaked 96-well filter mats using a 96-well Filtermate harvester, followed by at least three washes of cold wash buffer. Scintillation (MeltiLex) cocktail is melted onto dried filters and radioactivity is counted using a Wallac Trilux Microbeta (Perkin Elmer).

For receptor functional assays, Gs or Gi-coupled receptor activation was measured using a split-luciferase cAMP biosensor, GloSensor (Promega), and Gq-coupled receptor activation was measured as calcium flux using Fluo-4 Direct Dye (Invitrogen). HEKT cells (ATCC) transiently transfected or cells stably expressing the receptor were plated into 384-white (GloSensor) or black plates (Calcium flux) in DMEM containing 1% dialyzed FBS at least 6 hours to 24 hours before the assay. For GloSensor, the media was decanted and replaced with 20 μL drug buffer per well (HBSS, 20 mM HEPES, pH 7.4) containing GloSensor substrate. Cells were challenged with 10 μL of nuciferine or positive control (3X) to generate 16 point concentration curves and incubated for 15 minutes. For Gs-mediated cAMP accumulation, plates were read immediately. For Gi-mediated cAMP inhibition, 10 μL of isoproterenol (200 nM final concentration) was added to stimulate cAMP via endogenous β-adrenergic receptors and plates were read 15 minutes later. Luminescence was measured using Wallac TriLux Microbeta (Perkin Elmer) and luminescent counts per second (LCPS) was plotted. For Gq-mediated calcium flux, media was decanted and replaced with 20 μL drug buffer containing 2.5 mM probenecid and Fluo-4 dye and allowed to incubate for at least one hour at 37°C and 5% CO_2_ in a humidified incubator. Afterwards, 10 μL of nuciferine or positive control (3X) was added per well for 16 point concentrations and fluorescence was measured using FLIPR^TETRA^ (Molecular Devices). Maximum-fold increase over basal fluorescence was plotted. Results were analyzed using nonlinear regression to obtain EC_50_ using Graphpad Prism 5.0.

### 2.4 Dynamic light scattering to test for colloidal aggregation

Nuciferine was diluted into filtered water from a 10 mM stock in DMSO with 50 mM potassium phosphate, pH 7.0. Measurements were made at room temperature using a DynaPro MS/X (Wyatt Technology) with a 55 mW laser at 826.6 nm. The laser power was 100%, and the detector angle was 90° with samples run in duplicate.

### 2.5 AmpC β-lactamase assay counterscreen to test for colloidal aggregation

AmpC β -lactamase inhibition was measured in 50 mM potassium phosphate (pH 7.0) at room temperature as described. [21] Nuciferine was diluted from a 10 mM stock in DMSO and incubated with 1 nM AmpC for 5 minutes before the reaction was initiated by adding 92 μM CENTA substrate (Tydock Pharma; Modena, Italy). The final reaction volume was 1 mL. Change in absorbance was monitored at 405 nm for 2 minutes using an HP 8453 UV-Vis spectrophotometer. The assay was performed in duplicate in methacrylate cuvettes.

### 2.6 Vesicular monoamine transporter (VMAT2) study

*Cell culture*: HEK293 cells (ATCC) lines stably expressing hDAT or hDAT and hVMAT2 constructs were cultured at 37°C and 5% CO_2_ in DMEM with 10% FBS. All constructs were made in pcDNA3.1 (Life Technologies). hDAT and hVMAT2 expressing constructs contained a neomycin or zeocin resistance gene, respectively. Plasmids were transfected into HEK293 cells with Lipofectamine 2000. Stable cell lines were generated by repetitive rounds of limiting dilutions in selective media. Double stable cell lines were created by transfecting HEK-hDAT stable cells with the hVMAT2 construct and selecting for both plasmids with both neomycin and zeocin.

*Whole cell* ^*3*^*H-dopamine (DA) uptake*: Cells were plated into 48-well plates one day before uptake was performed. Cells were washed with 0.5 ml uptake buffer (4 mM Tris, 6.25 mM HEPES, 120 mM NaCl, 5 mM KCl, 1.2 mM CaCl_2_, 1.2 mM MgSO_4_, 5.6 mM D-glucose, 1.7 mM ascorbic acid, and 1 μM pargyline, pH 7.4). Cells were incubated with 225 μl uptake buffer with or without the indicated concentration of nuciferine for 15 minutes. After incubation, 25 μl uptake buffer containing ^3^H-DA and DA was added for a final concentration of 20 nM ^3^H-DA and 1 μM DA. Cells were incubated at 37°C for 20 minutes or for the time indicated. Nonspecific uptake was determined in the presence of 10 μM nomifensine. Uptake was terminated by aspirating uptake buffer and washing each well twice with 0.5 ml ice-cold uptake buffer. Cells were lysed in 0.1 N NaOH and transferred to vials containing 3 ml scintillation cocktail. Radioactivity was quantitated using a Beckman LS6500 counter. Data were analyzed in Graph Pad Prism 5.0.

*Vesicular* ^*3*^*H-DA Uptake*: Cells were plated in 10 cm dishes and grown to 100% confluency. Cells were washed with warm PBS without Ca^2+^ or Mg^2+^and resuspended in uptake buffer (25 mM HEPES, 100 mM potassium tartrate, 100 μM EDTA, 50 μM EGTA, pH 7.4). Cells were homogenized with a glass/Teflon homogenizer 30 times on ice. The homogenate was centrifuged at 8000xg for 8 minutes at 4°C. Protein content of the resulting supernatant was determined by BCA assay. Uptake assays utilized 100 μg of protein in complete uptake buffer (uptake buffer with 1.7 mM ascorbate, 2 mM Mg^2+^-ATP salt, pH 7.4) and 20 μM tetrabenazine (TBZ) to define specific uptake. Samples were incubated in uptake buffer with or without the indicated concentration of nuciferine for 10 minutes at 30°C followed by addition of 1 μM dopamine with a 2% tracer of ^3^H-DA. Samples were incubated for 5 minutes at 30°C with gentle shaking. The assay was terminated by addition of 5 ml ice-cold assay buffer before filtration through 0.5% PEI-soaked Whatman GF/F filters (Brandel Inc., Gaithersburg, MD). Filters were then placed in vials containing 3 ml scintillation fluid and counted using a Beckman LS6500. Data were analyzed in Graph Pad Prism 5.0.

### 2.7 Animals

University of North Carolina at Chapel Hill: The head-twitch response, locomotor activity, and catalepsy studies were run with male C57BL/6J mice bred at UNC. University of Arkansas for Medical Sciences: For DOI discrimination studies, adult male NIH Swiss mice weighing approximately 25 g were obtained from Harlan Laboratories (Indianapolis, IN, USA) and housed 3 mice per cage upon arrival. For PCP discrimination studies, adult male Sprague-Dawley rats weighing approximately 250 g were obtained from Harlan Laboratories (Indianapolis, IN, USA) and housed 3 rats per cage upon arrival. Virginia Commonwealth University: For clozapine substitution studies, adult male 129S2/SvHsd inbred mice were bred in-house. Duke University: adult male and female C57BL/6J mice, wild type (WT) mice, and dopamine transporter knockout (DAT-KO) mice were used in PPI experiments. The DAT mice were generated by heterozygous matings. DAT mice were group-housed in an environmentally-controlled room on a 14:10-h light/dark cycle (lights on 0800 h). The C57BL/6J, NIH Swiss, and 129S2/SvHsd mice and the Sprague-Dawley rats were housed in environmentally-controlled rooms on a 12:12-h light/dark cycle (lights on at 0700 hr). All experiments were conducted with approved protocols from the Institutional Animal Care and Use Committees of the university associated with each principal investigator: The University of North Carolina at Chapel Hill Institutional Animal Care and Use Committee, the Duke University Institutional Animal Care and Use Committee, the Virginia Commonwealth University Institutional Animal Care and Use Committee, and the University of Arkansas for Medical Sciences Animal Care and Use Committee. It should be noted that the range of species and breeds used in these studies was due to the “convenience and availability” nature of the collaboration, in which nuciferine was tested in the established experimental protocols of the collaborating laboratories dependent upon the availability of animals and openings in the experimental schedule.

### 2.8 Head-twitch responses

The head-twitch response procedure has been described elsewhere.[22–24] In brief, mice were injected with either nuciferine (1.0, 3.0, or 10.0 mg/kg, i.p.) or vehicle, n = 4 mice/condition. Fifteen minutes later, mice were injected with 1.0 mg/kg DOI (i.p.) and immediately placed in an observation chamber (new cage without bedding). Head-twitches (operationally defined as a rapid rotational jerk of the head that can be distinguished from species-appropriate grooming or scratching behaviors) were counted for 20 minutes in 5 minute bins. For the time-course study, mice were pretreated with 3.0 mg/kg nuciferine (i.p.) at 60, 45, 30, 15, or 0 minutes (co-injection) prior to the 1.0 mg/kg DOI (i.p.) injection, and head-twitches were counted as described above. In one experiment, mice (n = 4 per condition) were pretreated with an injection (s.c.) of 3.0 mg/kg nuciferine or vehicle 15 minutes prior to 1.0 mg/kg DOI injection (i.p.) and head-twitches were counted as described above. All experiments were performed by 3 observers, with 2 observers blinded to the experimental conditions which were evenly distributed. Power analyses were performed with the resulting data. The two highest doses of nuciferine tested (10.0 and 3.0 mg/kg), had 0.96 and 0.88 power to detect significance (α = 0.05). As these experiments were performed blinded and in distinct mice, further replication was not performed.

### 2.9 Open field activity

Locomotor activity was assessed in AccuScan activity monitors (41X41X30 cm; AccuScan Instruments, Columbus, OH) with photocells spaced at 1.52 cm as described. [22] In these monitors, the photocells create a grid of light beams, and breaks in the light beams (caused by the mouse) are recorded. The Accuscan software then calculates the total distance travelled (amongst other measurements) by analyzing the sequential order of beam breaks in the grid. Horizontal activity was measured as the total distance traveled in centimeters and was recorded in 5-minute bins. PCP-induced hyperlocomotor activity: mice (n = 16) previously acclimated to activity chambers were placed into the chambers for 15 minutes. Mice were then injected (i.p.) with either vehicle or 3 or 10 mg/kg nuciferine and returned to the chamber for 15 min. Subsequently, mice were injected with 6.0 mg/kg PCP (i.p.) and returned to the chamber for 90 minutes. Induction of AMPH-induced hyperlocomotor activity followed an identical protocol except locomotor activity was recorded for a total of 75 minutes and 3 mg/kg AMPH was used (i.p.).

### 2.10 Catalepsy procedure

Mice (n = 3) were initially injected (i.p.) with vehicle (0.9% saline/0.2% lactic acid), 10.0 mg/kg nuciferine, or 1.0 mg/kg haloperidol. Mice were placed upright on a 45° angled screen. The time required for the animal to move all four paws was scored in seconds (maximum of 5 min) and is reported as the latency to movement. An extended delay to move on the inclined screen test is indicative of drug-induced catalepsy. Power analyses were performed with the resulting data. The experiment had 100% power to detect a significant difference (α = 0.05) between nuciferine and haloperidol at the 60 minute timepoint.

### 2.11 DOI drug discrimination

Adult male NIH Swiss mice (n = 6) were trained to respond under an FR5 reinforcement schedule by presentation of evaporated milk in daily sessions using procedures similar to those previously described. [25] Mice were trained in drug discrimination via injection of saline (VEH) or 0.3 mg/kg R(-)-DOI presented in a pseudo-random order, with the constraint that no animal could receive the same injection for more than 3 consecutive sessions (1 session / day). Response assignments were counterbalanced across trials. Drugs were administered i.p. and pre-treatment time was 10 minutes. During each training session the overall response rate, overall distribution of responses on the drug-injection lever, and the distribution of responses on this same lever prior to delivery of the first reinforcer were analyzed. When animals reliably achieved a level of >85% correct responding prior to delivery of the first reinforcer over 3 consecutive sessions, a substitution test occurred the following day. During test sessions, a multiple component cumulative dosing procedure was used, and no responses were reinforced. Each component was terminated after the emission of five responses on either lever. Mice were then removed from the chamber, administered the next cumulative dose, and returned to the chamber. Ten minutes later, levers were re-extended into the experimental chamber. In this manner, four doses of drug could be tested over ~40 min in a single session. The distribution of responses between the two levers was expressed as a percentage of total responses emitted on the drug-appropriate lever. Response rate was calculated for each session by dividing the total number of responses emitted on both levers by the elapsed time prior to 5 responses on either lever. Nuciferine was administered 15 minutes prior to the first injection of DOI.

### 2.12 PCP drug discrimination

Adult male Sprague-Dawley rats (n = 5) were trained to respond under an FR20 schedule and were reinforced by presentation of food pellets in daily sessions using procedures similar to those previously described. [26] Rats were trained in drug discrimination via a pre-session injection of saline (VEH) or 3 mg/kg PCP chosen in a pseudo-random order (coin flip), with the same constraints, criteria, and dosing procedures as described above except that during test sessions, a given component of the cumulative dosing procedure was terminated after the emission of 20 responses on either lever. Drugs were administered i.p. and pretreatment time was 10 minutes. As described above for DOI, four doses of drug could be tested in a single ~40 min test session. Nuciferine was administered 15 minutes prior to the first injection of PCP.

### 2.13 Clozapine drug discrimination study

Adult male B6129 hybrid mice (n = 12) were trained to respond under a FR10 schedule and were reinforced by presentation of sweetened milk as described previously. [27] The drug and vehicle lever positions were counterbalanced between groups to control for olfactory cues. [28] All injections were given subcutaneously with a pre-session injection time of 30 minutes. Training occurred on a double alternation injection schedule with two days of VEH followed by two days of CLZ and repeated (VEH, VEH, CLZ, CLZ, VEH, VEH etc.). In order for a mouse to pass a training day it had to meet three criteria: (1) complete the first fixed ratio (FR) on the condition-appropriate lever, (2) at least 80% of the total responses were made on the condition-appropriate lever, and (3) at least 10 responses per minute were made during the session. Drug testing was conducted approximately two times per week with at least two training days in between. To be eligible for testing, mice were required to pass both a drug and vehicle training day consecutively. During drug substitution tests, animals were injected subcutaneously with nuciferine (0.1, 0.3, 1.0, 3.0, 10.0 mg/kg) and placed in the operant chamber after 30 minutes. Responses on both levers were reinforced.

### 2.14 Prepulse inhibition (PPI)

PPI of the acoustic startle response was conducted as described elsewhere [29] using SR-LAB chambers (San Diego Instruments, San Diego, CA). To determine whether nuciferine could ameliorate or normalize PPI, two separate experiments were conducted. In the first, C57BL/6J mice were administered VEH, 5, or 10 mg/kg nuciferine (i.p.) and returned to their home-cages for 15 min. Subsequently mice were treated with either VEH or 6 mg/kg PCP (i.p.) and placed into the PPI apparatus for a 5 min habituation prior to the onset of testing. In the second study, WT and DAT-KO mice were given VEH or 2.5, 5, or 10 mg/kg nuciferine or 2 mg/kg clozapine (i.p.) and returned to their home-cages. Fifteen min later DAT mice were habituated to the PPI apparatus for 5 min and testing began. The startle trials consisted of a 40 msec burst of 120dB white-noise; prepulse trials consisted of 20 msec prepulse stimuli that were 4, 8, or 12 dB above the white-noise background (64dB) and were followed 100 msec later by the 120dB acoustic startle stimulus. Non-stimulus or null trials consisted of the 64dB white-noise background. PPI responses were calculated as a percentage score for each prepulse intensity, where %PPI = [1–(prepulse trials/startle-only trials)]*100.

### 2.15 Statistics

The data are presented as means and standard errors of the mean (SEM) and data from the locomotor activity, head-twitch, and PPI studies were analyzed by SPSS statistical software (IBM Corp., Armonk, NY). Locomotor data were assessed with repeated measures ANOVA (RMANOVA) for within subject effects of time and for between subjects effects of treatment; head-twitch data were similarly assessed with RMANOVA. ANOVA and independent measures t-test were used to also examine treatment differences in motor activity. For the PPI experiment, the responses to the null and startle stimuli were analyzed by two-way ANOVA, whereas the PPI data were subjected to RMANOVA where the within subjects effect was prepulse intensity and the between subjects effects were treatment and, in the case of the DAT mice, genotype. All *post-hoc* analyses were by Bonferroni corrected pair-wise comparisons. A *p*<0.05 was considered significant. In the drug discrimination studies for each test session, mean (±SEM) percent responding on the drug-associated lever and the rate of responding (responses/sec) were calculated for each session component. Full substitution was operationally defined as >80% selection of the drug-associated lever, partial substitution was operationally defined as 40%-80% selection of the drug-associated lever, and no substitution was operationally defined as <40% selection of the drug-associated lever. Subjects failing to complete the response requirement in a given component were excluded from the data analyses, but their data were included in response rate calculations. Discrimination and response rate data were not normally distributed, so the Kruskal-Wallis one-way ANOVA on ranks was used to analyze data across dose, comparing 3 treatment conditions: 1) training drug alone, 2) training drug + 1.0 mg/kg nuciferine, and 3) training drug + 3.0 mg/kg nuciferine. Significant ANOVAs were followed by pair-wise multiple procedures using the Dunn's method (α = 0.05) to determine differences among means.

## 3. Results

### 3.1 Prediction, *in vitro* identification, and *in vitro* characterization of nuciferine

**Fig 3:**
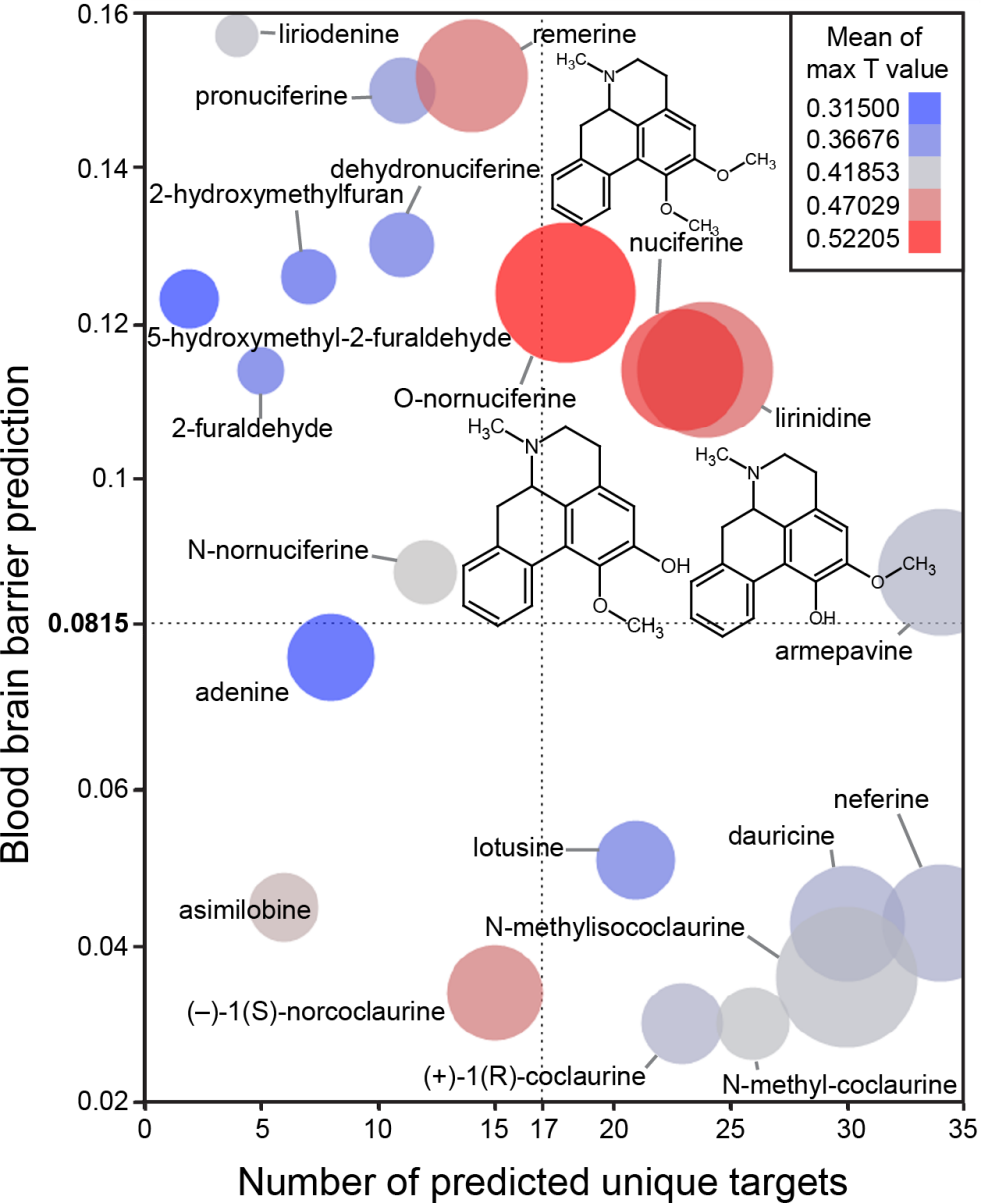
Bioinformatic predictions of lotus phytochemicals. X axis = number of unique predicted targets, as predicted by SEA. Each unique target included all affinity classes. Y axis = prediction of blood brain barrier penetration using the online blood-brain barrier prediction (BBB) server [17] (http://www.cbligand.org/BBB/). Higher values are predicted to pass the blood brain barrier. Size of circles = mean of -log10(e-value) for SEA-predicted targets. Larger circles indicate stronger confidence of predicted targets. Color of circles = Mean of max T values. Warmer colors (red) indicate better molecule-molecule matching, a second measure of prediction confidence. Y axis reference line (0.0815) and X axis reference line (17) are the average value for the world’s most widely prescribed psychiatric medications (aripiprazole and quetiapine).[74] These predictions suggest that nuciferine and its metabolites (O-nornuciferine, lirinidine) may be responsible for the psychotropic effects reported in humans. Chemical structures of nuciferine, O-nornuciferine, and lirinidine provided.

The *in silico* assessment of phytochemicals in *Nelumbo nucifera* (Fig 3) suggested that nuciferine and its metabolites are the most structurally similar to known compounds (Fig 3; color of circles). Additionally, nuciferine and its metabolites have high confidence protein-binding predictions (Fig 3; size of circles) and are predicted to cross the blood brain barrier (y axis). Finally, nuciferine is predicted to have a relatively large number of molecular targets (x axis).

Next we compared the polypharmacological profile of nuciferine with two atypical antipsychotic drugs (clozapine and aripiprazole) and the typical antipsychotic drug haloperidol (Fig 2).This convergence of predictions coupled with the potential utility of a compound with high polypharmacology suggested that nuciferine (Fig 1) would be the most pharmacologically interesting to investigate. Notable predictions from the SEA [13] are listed in Table 1, of which we were capable of testing 10 via the National Institute of Mental Health Psychoactive Drug Screening Program (PDSP).

**Table 1:** In silico and in vitro characterization of nuciferine.

SEA Predictions (in order of confidence)	In Vitro Affinity (nM)	Functional EC_50_ (nM)	Function Type
	Radioligand used		
D_1_ (5.4e-32)	752 [^3^H]SCH23390		
D_2_ (1.6e-25)	515 [^3^H]N-methyl Spiperone	65.07	Partial agonist
D_3_ (1.3e-12)	741 [^3^H]N-methyl Spiperone		
D_5_ (5.0e-13)	(neg) [^3^H]SCH23390	2600	Partial agonist, ~50%
VMAT2 (2.1e-13)	NA		
SK Channel (3.8e-11)	NA		
5-HT_1A_ (1.2e-11)	77 [^3^H]WAY100635	3230	Agonist
5-HT_5B_ (1.6e-7)			
5-HT_7_ (4.1e-4)	49.8 [^3^H]LSD	150	Inverse agonist
5-HT_2A_ (4.1e-6)	312 [^3^H]Ketanserin	478	Antagonist
**Unpredicted Hits**			
5-HT_2B_	41 [^3^H]LSD	1000	Antagonist
5-HT_2C_	60.5 [^3^H]Mesulergine	131	Antagonist
5-HT_6_	268 [^3^H]LSD	700	Partial agonist, 17.3%
D_4_	1387 [^3^H]N-methyl Spiperone	2000	agonist

Receptor targets are listed with their respective SEA prediction value (if available), followed by their competition binding affinity value (if available), followed by their functional EC_50_ value (if available) and the corresponding function type.

Information about cell lines for all assays can be found at http://pdsp.med.unc.edu/pdspw/binding.php.

**Fig 4:**
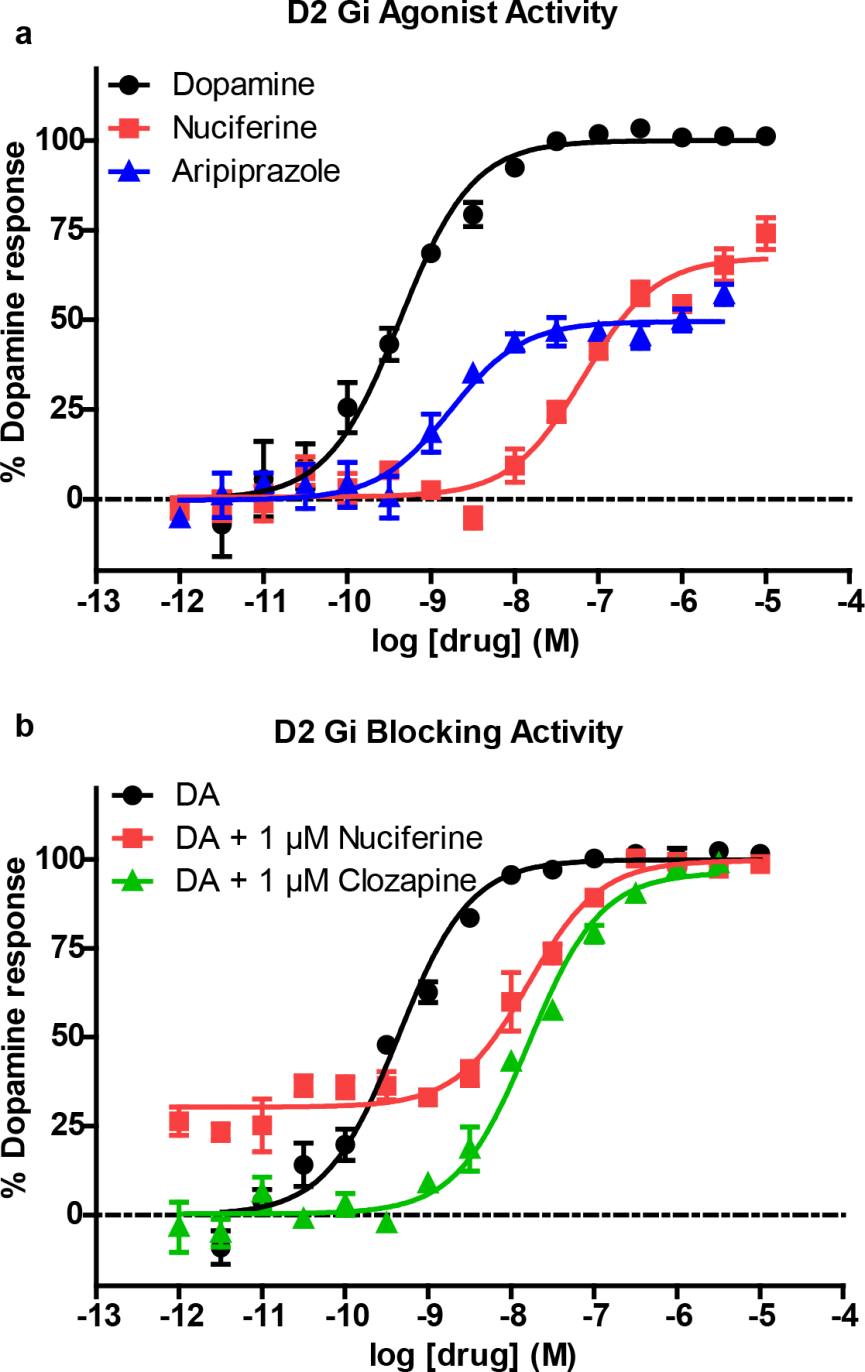
Nuciferine functional activity at the dopamine D_2_ receptor as measured via D_2_-mediated G_i_ signaling in HEKT cells. (a) Concentration-response curve of nuciferine (red) compared to dopamine (black) and aripiprazole (blue). (b) Concentration-response curve of dopamine (DA) in the presence of 1 μM nuciferine (red) or clozapine (green) showing rightward-shift of DA indicative of competitive antagonist activity. The data represent concentration-response curves of normalized data with respect to dopamine performed in triplicate (mean +/- s.e.m.; n = 3).

The PDSP *in vitro* affinity screening (Tables 1 and 2) revealed a total of 13 receptors with affinities less than 1 μM, and 21 receptors with affinities less than 10 μM. SEA successfully predicted 7 out of 13 G protein-coupled receptors that were determined by the PDSP to have K_i_ values of less than 1 μM. Functional studies (Table 1) indicate that nuciferine shows appreciable potency as a D_2_ partial agonist (EC_50_ = 64 nM), as a 5-HT_7_ inverse agonist (EC_50_ = 150 nM), and as a 5-HT_2C_ antagonist (IC_50_ = 131 nM). [30] Nuciferine was a partial agonist at D_2_ receptors with an activity (E_max_ = 67% of dopamine, Fig 4A) similar to aripiprazole (E_max_ = 50% of dopamine).[19, 31] In line with its partial agonist activity, nuciferine inhibited dopamine-induced activation of G_i_ (Fig 4B) with a potency similar to clozapine (nuciferine K_B_ = 62 nM; clozapine K_B_ = 20 nM) as determined via Schild regression analysis. [32] Also similar to clozapine, nuciferine exhibits 5-HT_2A_ antagonist activity (IC_50_ = 478 nM) and 5-HT_7_ inverse agonist activity (IC_50_ = 150 nM), [33] and may effectively antagonize 5-HT_6_ receptors as a low efficacy partial agonist (EC_50_ = 700 nM, 17.3% maximal effect). [2, 34] Finally, nuciferine exhibited micromolar potency as a 5-HT_2B_ antagonist (IC_50_ = 1 μM), a D_4_ agonist (EC_50_ = 2 μM), a D_5_ partial agonist (EC_50_ = 2.6 μM, 50% maximal response), and a 5-HT_1A_ agonist (EC_50_ = 3.2 μM).

**Table 2:** In vitro affinity findings lacking functional measurements.

PDSP Hits Without Functional Assay	In vitro Affinity (nM) Radioligand used
5-HT_1D_	518 [^3^H]5-CT
α_1A_	1386 [^3^H]Prazosin
α_1B_	1995.3 [^3^H]Prazosin
α_1D_	818 [^3^H]Prazosin
α_2A_	1153.5 [^3^H]Rauwolscine
α_2B_	686.8 [^3^H]Rauwolscine
α_2C_	692.8 [^3^H]Rauwolscine
β_1_-AR	7149 [^3^H]CGP12177
β_3_-AR	1103 [^3^H]CGP12177
DOR	10000 [^3^H]DADLE
H_2_	1662 [^3^H]Cimetidine
MOR	9549.00 [^3^H]DAMGO
V1A	10000 [^3^H]Vasopressin
5-HT_5A_	1113 [^3^H]LSD

Receptor targets not predicted by SEA but identified by in vitro competition binding assays for which functional assays are not available. The following receptors assayed in the PDSP screen had no observed binding of nuciferine. Receptors are listed with their associated radioligand in (). Sets of receptors using the same radioligand are indicated in []: [5-HT1B, 5-HT1E]([^3^H]5-CT), 5-HT3 ([^3^H]GR65630), β2-AR ([^3^H]CGP12177), BZP Rat Brain Site ([^3^H]Flunitrazepam), D5([^3^H]SCH23390), DAT([^3^H]WIN35428), GabaA ([^3^H]Muscimol), H1 ([^3^H]Pyrilamine), H3 ([^3^H]α-methylhistamine), H4 ([^3^H]Histamine), KOR ([^3^H]U69593), NMDA PCP site ([^3^H]MK801), [M1, M2, M3, M4, M5]([^3^H]QNB or [^3^H]NMS), NET ([^3^H]Nisoxetine), Oxytocin([^3^H]Oxytocin), SERT ([^3^H]Citalopram), Sigma 1 ([^3^H]Pentazocine), Sigma 2 ([^3^H]DTG), [V1B, V2]([3H]Vasopressin).

### 3.2 Assessment of aggregator properties

The predominantly low-potency antagonist activity of nuciferine suggested that nuciferine could be functioning as a colloidal aggregator, a well-known mechanism of promiscuous activity in early discovery, such as high-throughput screening. [35, 36] Recent studies have shown this mechanism can affect membrane-bound receptors such as GPCRs [37] in cell-based screening. At concentrations relevant to this study (< = 10 μM), dynamic light scattering determined that nuciferine did not scatter light above background in aqueous buffer, suggesting colloidal aggregates of nuciferine are not formed at this concentration. Consistent with this, nuciferine did not inhibit AmpC β-lactamase at these concentrations; AmpC is a counter-screening enzyme widely used to test for colloidal aggregation [35, 36, 38] (data not shown). The lack of particle formation by light scattering and the lack of inhibition of the orthogonal counter-screening enzyme supports the idea that nuciferine is not acting promiscuously via colloidal aggregation in the assays described here.

### 3.3 Dopamine transport by DAT and VMAT2

**Fig 5:**
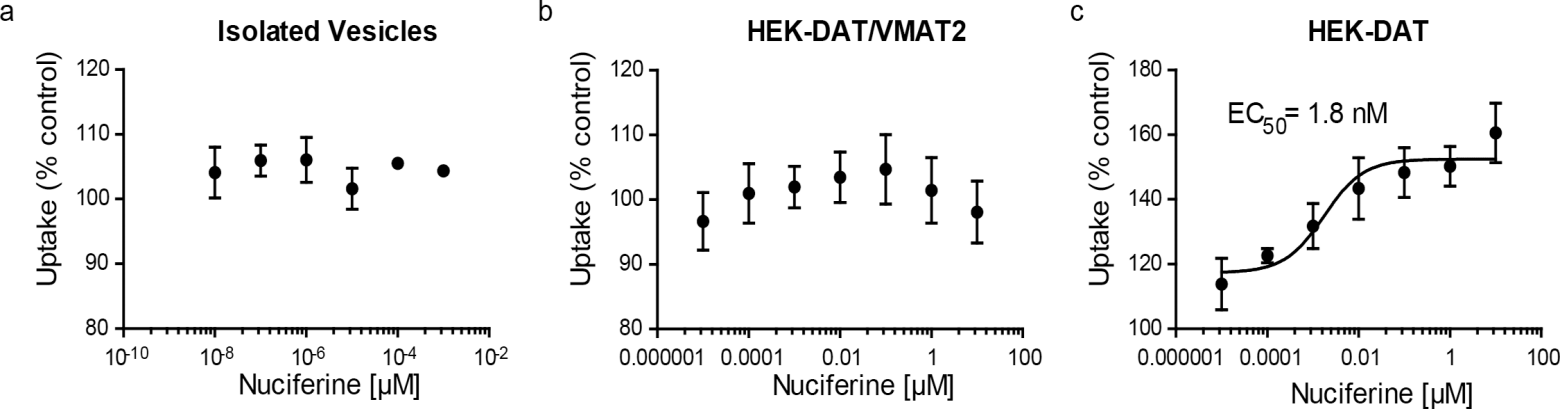
DAT modulation by nuciferine. (a) Concentration-response curves of nuciferine on vesicular uptake in isolated vesicles, (b) concentration-response curves of nuciferine on vesicular uptake in HEK cells transfected with DAT and VMAT2, (c) concentration response curves of nuciferine on vesicular uptake in HEK cells transfected with DAT. Data are presented as a percentage of the response to vehicle control. The data represent concentration-response curves of normalized data with respect to vehicle performed in triplicate (mean +/- s.e.m.; n = 3).

SEA predicted that nuciferine may interact with the vesicular monoamine transporter-2 (VMAT2). To determine if nuciferine affects uptake at VMAT2, we conducted dopamine uptake experiments in vesicles isolated from HEK cells overexpressing VMAT2 and found no effect of nuciferine on DA uptake (Fig 5A). We also tested whether nuciferine affected uptake in whole-cell uptake studies in HEK cells expressing DAT and VMAT2 or DAT alone as a control, as described elsewhere. [39] In whole cells expressing DAT and VMAT2, the total capacity of the cell to take up and store dopamine is measured. This has a DAT-mediated and a VMAT2-mediated component, as previously demonstrated.[39] Comparison of the effects of compounds in these two cell types, as well as in isolated vesicles, aids in separating out these two components of uptake in whole cells. Nuciferine pre-treatment had no effect in HEK-DAT/VMAT2 cells (Fig 5B). Counter to predictions, in the HEK-DAT control cells, nuciferine pre-treatment increased uptake in HEK-DAT cells by 60% over the vehicle control (Fig 5C; EC_50_ = 1.8 nM).

### 3.4 DOI-induced head-twitch response (HTR)

**Fig 6:**
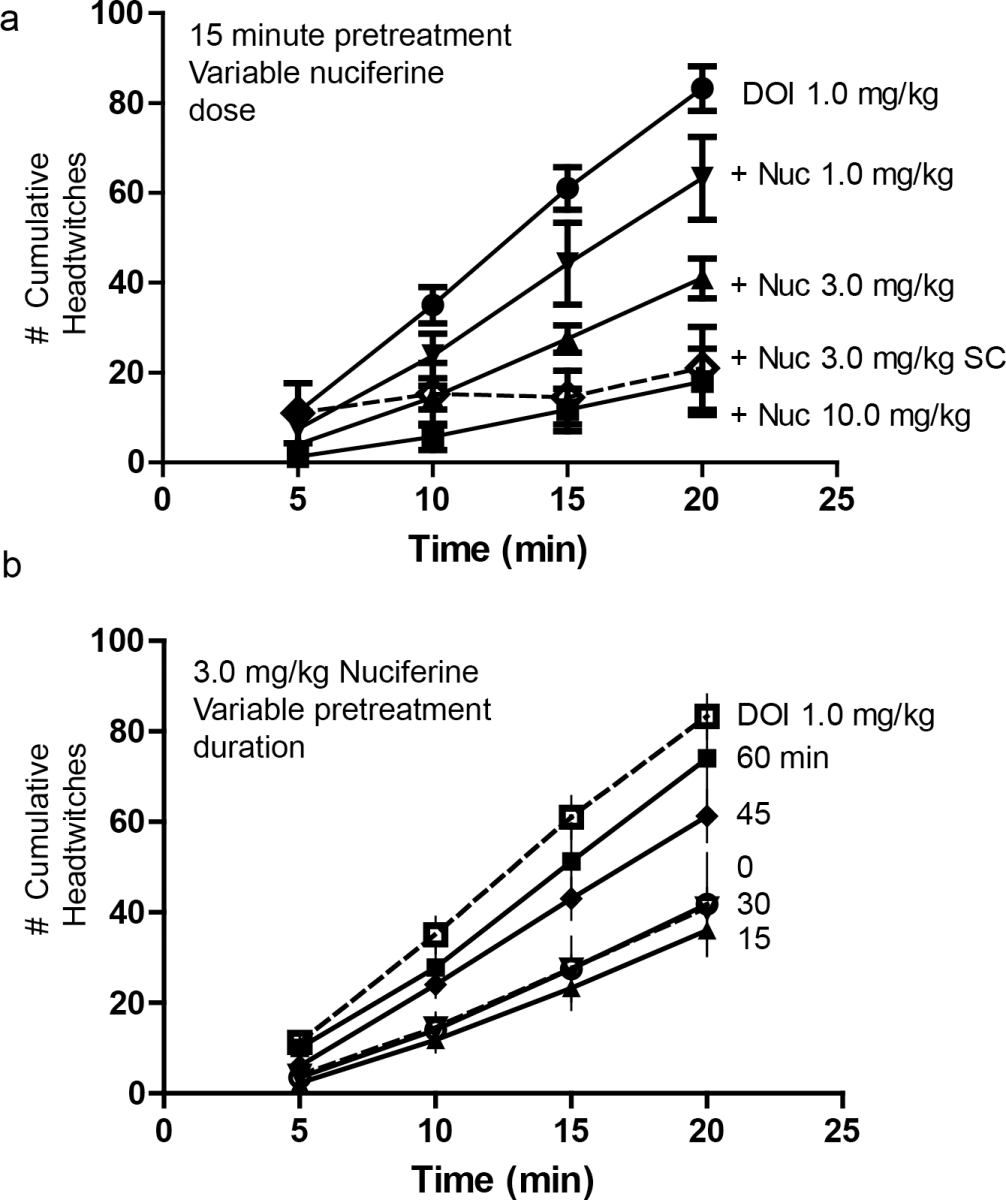
Inhibition of the 5-HT_2A_ mediated, DOI-induced head-twitch response by nuciferine. (a) Dose response curves of a 15-minute nuciferine pretreatment on the 1.0 mg/kg DOI-induced head twitch response. Solid lines indicate intraperitoneal administration of nuciferine. Dashed line indicates subcutaneous administration of nuciferine. Dose of nuciferine indicated to right of its associated data line. (b) Effect of nuciferine pretreatment time on the suppression of the DOI-induced head-twitch response. All pretreatments were administered intraperitoneally. DOI-alone condition provided as reference. Pretreatment time indicated to right of its associated data line. Data are presented as the mean number of cumulative head-twitches (y axis) at the given time point (x axis) (mean +/- s.e.m.; n = 3).

Mice pretreated with nuciferine (1.0, 3.0 and 10.0 mg/kg, i.p. or 3 mg/kg, s.c.;15 min prior to DOI) showed a dose-dependent inhibition of the head-twitch response produced by 1.0 mg/kg DOI during the course of testing (Fig 6A). Bonferroni corrected pair-wise comparisons indicated that regardless of route of injection, nuciferine attenuated DOI-induced head-twitches at 10, 15 and 20 min (*p*s<0.05). When the different doses of nuciferine were examined, a RMANOVA revealed a significant effect of time [F_[__3__,__36__]_ = 232.725, *p*<0.0001], and a significant time by treatment interaction [F_[__9__,__36__]_ = 16.170, *p*<0.0001]. Bonferroni comparison results indicated that 3 and 10 mg/kg nuciferine decreased head-twitches at 5, 10, 15 and 20 min (*p*s<0.05) relative to DOI-treated mice. Differences in responses to the 3 and 10 mg/kg doses were not statistically significant (Fig 6A). By comparison, 1 mg/kg nuciferine exerted no statistical effect on the DOI induced head-twitches.

The time-course of 3 mg/kg nuciferine pre-treatment (i.p.) indicated that suppression of the DOI-induced head-twitches was most pronounced when the interval between the nuciferine and DOI injections was 15 min (Fig 6B). When the interval between nuciferine and DOI injections was varied, a RMANOVA found the main effect of time [F_[__9__,__54__]_ = 334.913, *p*<0.001] and the time by pre-treatment interval [F_[__15__,__54__]_ = 5.838, *p*<0.001] to be significant. Bonferroni analyses indicated that mice treated 45 or 60 min prior to DOI injection failed to show significant alterations in head-twitches compared to DOI-treated animals (Fig 6B). By contrast, mice treated 15 or 30 min prior to the DOI injection all had statistically significant (*p*<0.05) reductions in the numbers of head-twitches compared to mice given DOI at the 5, 10, 15, and 20-min sampling times. Moreover, nuciferine pretreatment at 15 min produced the most profound overall reductions in head-twitches relative to animals pretreated at 45 and 60 min and measured at 15 and 20 min sampling times, all of which were statistically significant (*p*<0.05). Collectively, these data demonstrate that nuciferine can antagonize DOI-induced head twitches and that these effects are time-dependent with the 15 min interval between nuciferine and DOI injection being the most effective.

### 3.5 Drug discrimination

**Fig 7:**
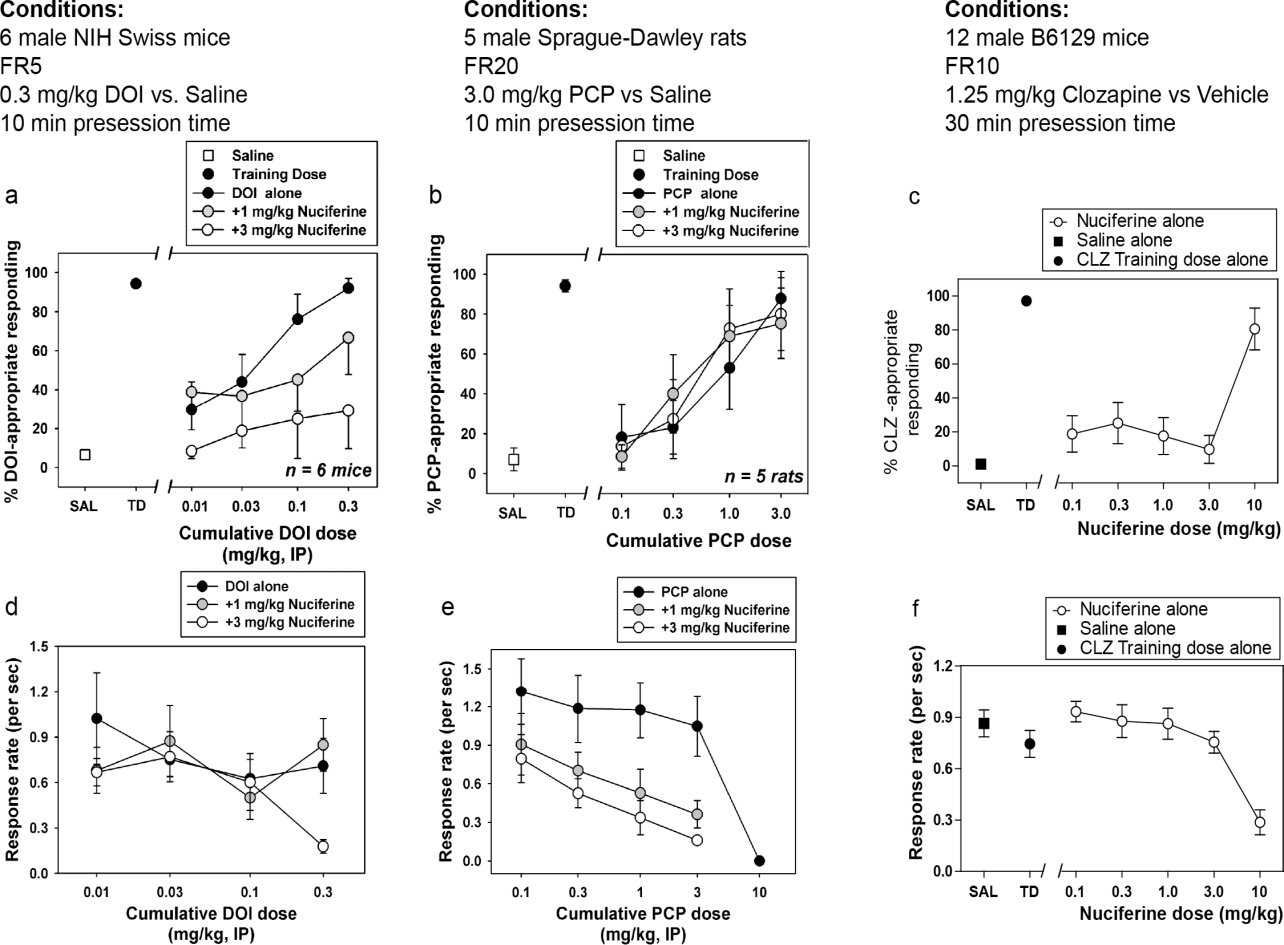
Drug Discrimination studies of nuciferine. (a,d) Nuciferine blocks the discriminative stimulus of DOI at doses that do not affect the rate of responding. (b,e) Nuciferine does not block the discriminative stimulus of PCP at any dose tested. (e,f) Nuciferine substitutes for clozapine (solid circles) but only at a dose that suppresses response rate (empty circles). Data are presented as % training-drug appropriate responding and response rate per second.

Dose-dependent generalization for the DOI training dose was observed when cumulative doses were administered alone, with cumulative doses of 0.1 and 0.3 mg/kg producing full substitution (Fig 7A). In the presence of 1.0 mg/kg nuciferine, however, cumulative DOI doses only produced partial substitution, whereas in the presence of 3.0 mg/kg nuciferine, no cumulative dose of DOI substituted for the training dose, up to a dose that profoundly suppressed responding (Fig 7D). Although the overall ANOVA was significant (p<0.001), no within-dose pairwise comparisons reached statistical significance. Dose-dependent generalization for the PCP training dose was observed when cumulative doses were administered alone, with a cumulative dose of 3.0 mg/kg producing full substitution. In the presence of 1.0 or 3.0 mg/kg nuciferine, cumulative PCP doses produced similar substitution to PCP alone (Fig 7B). Although the overall ANOVA was significant (p<0.05), no within-dose pairwise comparisons reached statistical significance. In the clozapine-trained animals, a dose-dependent substitution for 1.25 mg/kg clozapine was seen at 10.0 mg/kg nuciferine (80.63% drug lever responding), with an ED_50_ value of 5.42 mg/kg (95% CI 3.09–9.48 mg/kg) while the lower doses tested (0.1 mg/kg–3.0 mg/kg) failed to produce substitution for clozapine’s discriminative cue (Fig 7C). In addition to a high percentage of responding on the clozapine-appropriate lever, 10.0 mg/kg nuciferine also produced significant rate suppression as compared to vehicle control points (p < 0.001) (Fig 7F).

### 3.6 Locomotor activity

**Fig 8:**
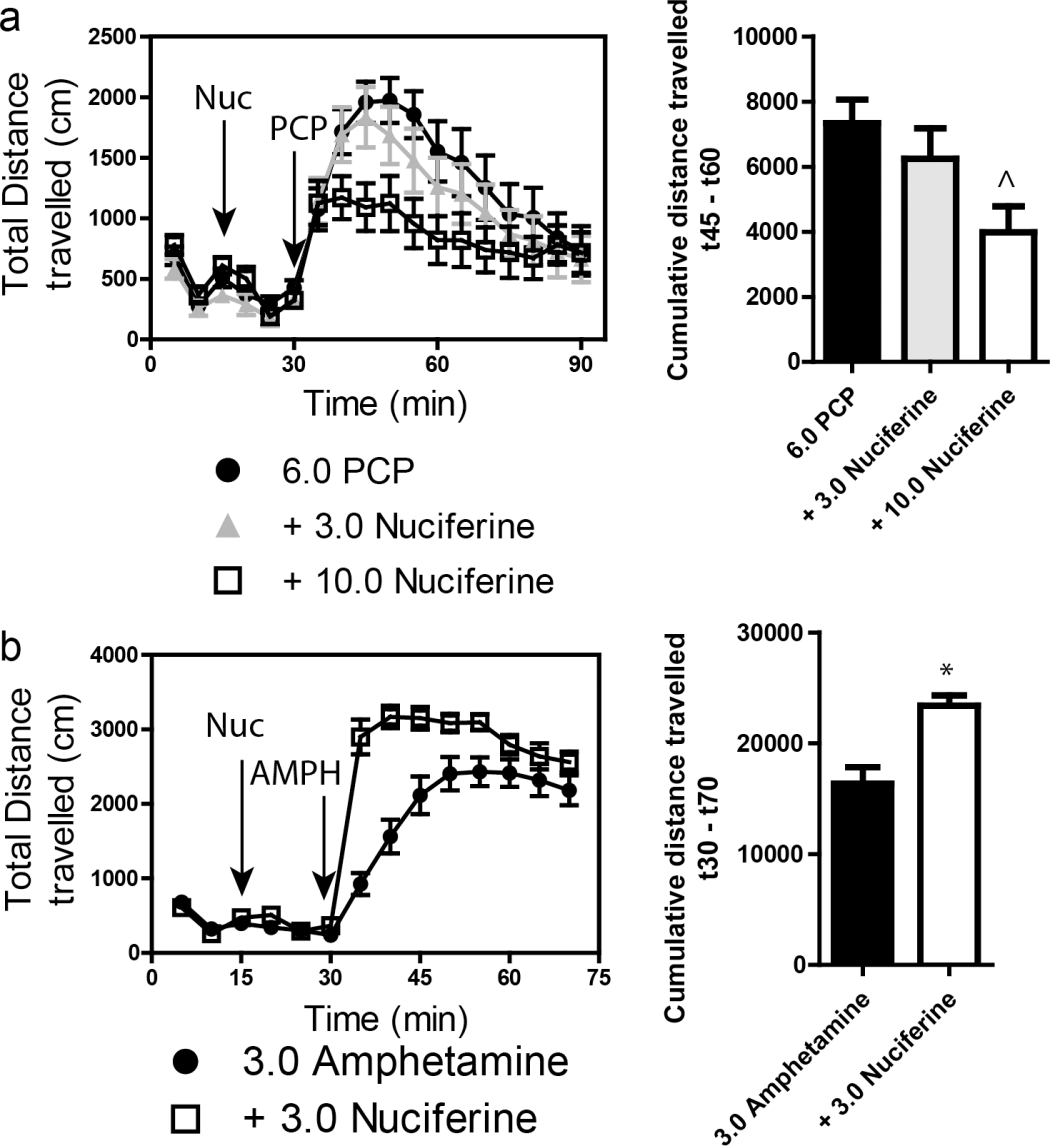
Locomotor studies of nuciferine. (a) Nuciferine suppresses the PCP-induced hyperlocomotor response. Data are presented as total distance travelled in 5-minute bins (left) and as cumulative distance travelled between minute 45 and minute 60 (right). N = 18 mice; ^p<0.05, compared to PCP group. (b) Nuciferine (3.0 mg/kg, 15 minute pretreatment) enhances the hyperlocomotor effect of amphetamine (3.0 mg/kg) administration. N = 14 mice; * < 0.001 compared to Amphetamine group. Data are presented as above.

Stimulation of locomotor activity by 6.0 mg/kg PCP was significantly blocked by 10.0 mg/kg, but not by 3.0 mg/kg, nuciferine (Fig 8A. left panel). RMANOVA revealed a significant effect of time [F_[__17__,765]_ = 30.553, *p*<0.001], and a significant time by treatment interaction [F_[__34__,765]_ = 1.997, *p*<0.010]. Bonferroni corrections indicated that during the 0–30 min interval locomotion in all groups was similar. As expected, PCP stimulated locomotor activity from 35–90 min compared to baseline at 0–30 min (*p*s<0.001). Treatment with 3 mg/kg nuciferine prior to PCP failed to significantly alter the PCP-induced hyperlocomotion, whereas the 10 mg/kg nuciferine significantly depressed this locomotion compared to PCP-treated mice at 45–60 min (*p*<0.05). Nevertheless, the reductions in PCP-stimulated activity by 3 and 10 mg/kg nuciferine were not statistically different. When the results were presented as cumulative distance traveled between 45 and 60 min of testing, dose-dependent decreases in PCP-stimulated activity could be seen (Fig 8A, right panel). ANOVA found a significant effect of treatment [F_[__2__,__47__]_ = 4.323, *p*<0.05] and Bonferroni tests confirmed that 10 mg/kg nuciferine significantly reduced locomotion compared to PCP (*p*<0.05). The 3 mg/kg dose decreased activity, but it was not significantly different from the PCP alone or the 10 mg/kg plus PCP group.

A second set of mice was used to assess the effects of 3 mg/kg nuciferine pre-treatment on amphetamine induced hyperlocomotion (Fig 8B, left panel). A RMANOVA revealed a significant effect of time [F_[__13__,390]_ = 177.243, *p*<0.001] and a significant time by treatment interaction [F_[__13__,390]_ = 14.625, *p*<0.001]. Bonferroni corrected comparisons found that activities between the two group at 0–30 min were not statistically different. All animals showed a significant increase in activity following amphetamine treatment compared to their baseline activities (*p*<0.001). Those mice given nuciferine prior to amphetamine treatment showed significantly higher motor activity between 35–50 min compared to those given amphetamine alone (*p*<0.05); however, responses after this time were not significantly differentiated. When the distance travelled in the open field for the 40 min following amphetamine treatment was aggregated, mice given nuciferine followed by amphetamine had heightened activity compared to those animals given amphetamine alone (Fig 8B, right panel) [t_[__1__,__30__]_ = 4.014, *p*<0.001]. Together, these findings indicate that while nuciferine is capable of attenuating the hyperlocomotion induced by PCP, amphetamine induced hyperlocomotion is exacerbated by the compound.

### 3.7 Pre-pulse inhibition

**Fig 9:**
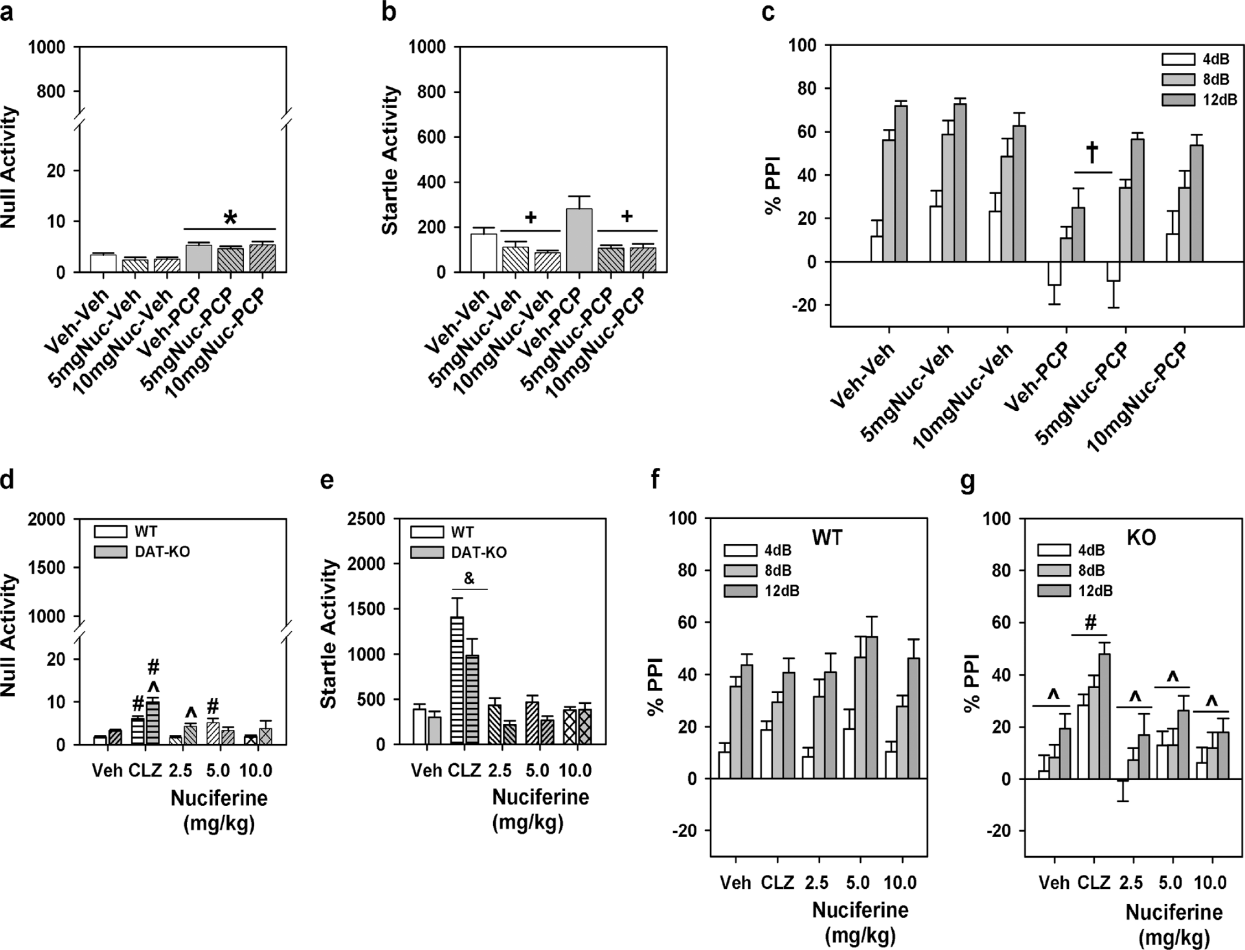
PPI responses to nuciferine in mouse models of hypoglutamatergia and hyperdopaminergia. Nuciferine rescued PPI in the former, but not in the latter model. (a-c) Null activity (a), startle activity (b), and PPI (c) for C57BL/6J mice treated with vehicle (Veh), 5 or 10 mg/kg nuciferine (Nuc), and/or phencyclidine (PCP). (d-g) Null activity (d), startle activity (e), and PPI for wild-type (WT) (f) and dopamine transporter knockout (DAT-KO) mice (g) given Veh, 2 mg/kg clozapine (CLZ) or 2.5–10 mg/kg Nuc. N = 8–17 mice/group in the C57BL/6J experiment; *p<0.05, compared to Veh/Nuc groups; +*p*<0.05, compared to the Veh and PCP groups; †*p*<0.05, compared to all other groups. N = 9–17 mice/genotype/treatment in the DAT experiment; ^*p*<0.05, WT versus KO within dose; #*p*<0.05, dose effect within genotype; &*p*<0.05, overall drug effect regardless of genotype.

For the experiment with C57BL/6J mice a two-way ANOVA was applied with two levels of treatment: PCP (vehicle or VEH and PCP) and nuciferine (VEH and the two doses of nuciferine) constituted the 6 groups. A two-way ANOVA for null activity identified a main effect of PCP treatment [F_(1,45)_ = 31.70, *p*<0.001], but the nuciferine treatments and the PCP by nuciferine interaction was not significant. Although Bonferroni corrected pair-wise comparisons noted that null activity was higher in the PCP-treated groups than in the respective vehicle/nuciferine groups (*p*<0.001), this activity was still less than 7% of the startle responses in the PCP-treated group (Fig 9A). A two-way ANOVA for startle responses also observed a significant main effect of nuciferine treatment [F_(2,45)_ = 13.22, *p*<0.001]; the PCP treatment effect and the PCP by nuciferine interaction was not significant. Bonferroni *post-hoc* tests reported that startle responses for the 5 and 10 mg/kg nuciferine-treated groups were lower than those of the vehicle-treated groups (*p*<0.001; Fig 9B). RMANOVA for PPI revealed the within subjects main effects for prepulse intensity was significant [F_(2,90)_ = 93.16, *p*<0.001]; the prepulse-intensity by PCP treatment, prepulse-intensity by nuciferine treatment, and the prepulse-intensity by PCP by nuciferine treatment interactions were not significant (Fig 9C). Bonferroni tests demonstrated that the response to the 4dB prepulse was lower than that for the 8 and 12dB prepulse responses (*p*<0.001) and that the 8dB response was lower than that for the 12dB response (*p*<0.001). Regardless of prepulse intensity, the between subjects main effects for the nuciferine [F_(2,45)_ = 3.43, *p*<0.05] and PCP treatments [F_(1,45)_ = 33.85, *p*<0.001], as well as the nuciferine by PCP treatment interaction, were significant [F_(2,45)_ = 3.31, *p*<0.05]. Decomposition of the interaction observed that PPI in the vehicle-vehicle, 5 mg/kg nuciferine-vehicle, and 10 mg/kg nuciferine-vehicle groups were not differentiated from each other, but that PPI in the PCP-vehicle group was suppressed relative to these controls (*p*<0.001). Despite this fact, mice given nuciferine prior to PCP had higher PPI than the PCP-vehicle groups (*p*<0.05) and the 10 mg/kg nuciferine-PCP group was not statistically different from the respective control. Thus 10/mg/kg nuciferine rescued the PCP-disrupted PPI.

Analyses of responses from the DAT KO mice presented a different picture. A two-way ANOVA for null activity observed significant genotype [F_(1,102)_ = 12.410, *p*<0.005] and treatment effects [F_(4,102)_ = 18.516, *p*<0.001] and a significant genotype by treatment interaction [F_(4,102)_ = 3.956, *p*<0.01]. Bonferroni corrections demonstrated that null activity in DAT-KO mice was higher than that in WT mice with clozapine *(p*<0.005) and 2.5 mg/kg nuciferine (*p*<0.01) (Fig 9D). Null activity in WT mice to clozapine and 5 mg/kg nuciferine were significantly enhanced relative to the vehicle control (*p*<0.005), whereas null activity in DAT-KO mice to clozapine and all doses of nuciferine were significantly augmented compared to vehicle group (*p*<0.001). A two-way ANOVA for startle responses revealed significant main effects of genotype [F_(1,102)_ = 9.227, *p*<0.005] and treatment [F_(4,102)_ = 28.673, *p*<0.001] (Fig 9E). Regardless of genotype, clozapine-treated mice had higher startle responses than all other groups (*p*<0.001). For PPI a RMANOVA found significant within-subject effects of prepulse intensity [F_(2,204)_ = 60.090, *p*<0.001] and a significant prepulse intensity by genotype interaction [F_(2,204)_ = 10.479, *p*<0.001]. Bonferroni tests demonstrated prepulse dependency in WT animals where inhibition at each prepulse intensity was significantly different from each other (*p*<0.001) (Fig 9F). By contrast, in DAT-KO animals responses to the 12 dB prepulse were higher than those to the 4 and 8 dB prepulses (*p*<0.005), which were not significantly distinguished from each other (Fig 9G). The between subjects test discerned significant genotype [F_(1,102)_ = 25.246, *p*<0.001] and treatment effects [F_(4,102)_ = 4.238, *p*<0.005] and a significant genotype by treatment interaction [F_(4,102)_ = 3.288, *p*<0.05]. Bonferroni comparisons found that PPI was significantly lower for DAT-KO than WT mice in the groups given vehicle or any dose of nuciferine (*p*<0.05). Clozapine significantly enhanced PPI in DAT-KO mice relative to the vehicle and nuciferine treatments (*p*<0.05). Together, these data show that PPI is intact in WT animals and it is not affected by either clozapine or nuciferine. By contrast, PPI is deficient in DAT-KO mice and it was restored by clozapine but not by nuciferine.

### 3.8 Catalepsy

Nuciferine (10.0 mg/kg) did not produce catalepsy at any of the time-points examined (Table 3).

**Table 3:** Cataleptic properties of nuciferine.

Post-injection timepoint (min)	Vehicle	Nuciferine10.0 mg/kg	Haloperidol1.0 mg/kg
**30**	3 ± 2.08 s	2.3 ± 2.3 s	270 ± 30 s
**60**	2.67 ± 1.45 s	4.7 ± 2.6 s	300 ± 0 s

Nuciferine did not cause a latency to move in the inclined grid test compared to haloperidol at the doses and times tested.

## 4. Discussion

Here we present an *in vitro* and *in vivo* characterization of nuciferine, an alkaloid found in *Nelumbo nucifera* and *Nymphaue caerulea* lotus plants. Our primary finding was that nuciferine has a pharmacological profile similar but not identical to some antipsychotic drugs (especially aripiprazole) and that nuciferine performed as an antipsychotic-like drug in some animal models predictive of antipsychotic drug-like actions. Here, we discuss these findings in the context of antipsychotic pharmacology and animal models of antipsychotic efficacy.

### 4.1 Molecular characterization

The *in silico* prediction and *in vitro* characterization of nuciferine revealed a molecular affinity profile containing receptors known to be modulated by established antipsychotic drugs. Within the set of 13 receptors with <1 μM affinity, nuciferine showed greatest affinity at the serotonergic receptors and overall it exhibited a molecular profile with multiple entities implicated in clinical antipsychotic efficacy: 5-HT_7_ [29, 34, 40, 41], 5-HT_6_ [34], 5-HT_2A_ [5], 5-HT_1A_ [42], 5-HT_1D_ [43], D_2_ [44], D_1_, D_3_, D_4_, D_5_ [45], α_2B_, and α_2C_ [46]. The preponderance of low affinity antagonism led us to test whether nuciferine forms colloidal aggregates. Parenthetically, colloidal aggregation is a phenomenon that affects molecular pharmacological screening efforts and has not been appreciated until relatively recently.[47] Compounds that form colloidal aggregates can present unique and irrelevant behavior *in vitro* [48], and we avoided these confounds by demonstrating that nuciferine does not form aggregates at concentrations up to 10 μM. Of the non-GPCR targets that SEA predicted, SK channels have been shown to bind antipsychotic compounds, [49] while VMAT2 is involved in monoaminergic neurotransmission and has long been proposed as a target for antipsychotic drug activity.[50, 51] It is also interesting to note that nuciferine does not bind to any muscarinic receptors. Muscarinic antagonists are prescribed to prevent or treat extrapyramidal side effects of antipsychotics. [52–54] During the course of our investigation, an independent group determined that nuciferine functions as a 5-HT_2A_ antagonist [55], corroborating our findings at this receptor.

With respect to antipsychotic efficacy, the D_2_ receptor is a desired druggable target with all approved antipsychotic drugs having potent interactions with this target.[30] In addition to D_2_ receptor affinity, however, it is also known that partial agonists have antipsychotic efficacy, such as aripiprazole.[19, 31] Therefore, we examined the D_2_ functional properties of nuciferine in comparison to aripiprazole and found that nuciferine exhibited a similar degree of partial agonist activity compared to aripiprazole, suggesting that nuciferine could exhibit antipsychotic drug-like properties, albeit with lower potency. The ability to block or antagonize DA-stimulated D_2_ activity may be a better predictor of antipsychotic efficacy, especially in the case of D_2_ partial agonists such as aripiprazole with low intrinsic efficacy *in vitro*,[56] which may act as D_2_ antagonists *in vivo*. [57] To examine nuciferine’s antagonist activity, we chose to measure nuciferine’s ability to block DA-stimulated D_2_ activation using a Schild regression analysis.[32] Results indicated that nuciferine can antagonize DA-stimulated cAMP inhibition with a potency similar to clozapine. Clozapine, one of the most effective atypical antipsychotics, possesses lower affinity for the D_2_ receptor compared to typical antipsychotics, [5] and therefore it is conceivable that just a moderate degree of D_2_ antagonism is needed for antipsychotic efficacy, given that clozapine also possesses potent 5-HT_2A_ antagonism, 5-HT_1A_ partial agonist activity, and 5-HT_7_ inverse agonism, which nuciferine also possesses. In summary, nuciferine shares two properties of antipsychotic efficacy at the D_2_ receptor, namely partial agonist activity at the D_2_ receptor similar to aripiprazole, and antagonist activity with moderate affinity comparable to clozapine.

There were notable discrepancies within our study across paradigms and compared to previously published findings. For instance, the SEA predicted one entity (dopamine D_5_ receptor) that was not detected in the PDSP binding affinity assay but was detected in the functional assay. The micromolar efficacy of nuciferine at the D_5_ receptor as measured via functional assays is consistent with the low binding affinity, due to the fact that the antagonist radioligand [^3^H]SCH 23390 labels inactive states of the receptor. Additionally, the dopamine transporter (DAT) was neither predicted using the SEA nor was it detected in the PDSP screen, yet it was determined that nuciferine modulates the DAT *in vitro* using a functional assay, again suggesting that this compound may be modulating the DAT at a site other than that occupied by the radioligand [^3^H]WIN35428. Interestingly, our behavioral results with amphetamine in the open field also support the notion that nuciferine interacts with DAT. It was previously reported that nuciferine inhibits the hyperlocomotor effect of amphetamine [4], but in our study we observed an increase in hyperlocomotor activity following nuciferine pretreatment. Our methods differed substantially from those of Bhattacharya et al. [4] who used a considerably higher dose range (25–100 mg/kg) of nuciferine, compared to our lower dose range (1–10 mg/kg across the study with 3.0 mg/kg nuciferine in the amphetamine experiment). These discrepancies support the use of orthologous assays in screening efforts to fully elucidate a chemical’s pharmacology.

The discovery of DAT modulation occurred serendipitously during the verification of the SEA VMAT2 prediction due to the nature of the experimental system. In these experiments, nuciferine caused an increase in uptake in cells expressing DAT but showed no effect in cells expressing both DAT and VMAT2. Direct measurement of uptake in isolated vesicles failed to detect direct modulation of VMAT2 by nuciferine. The difference between the results of the DAT and DAT/VMAT2 cells suggests that nuciferine may be indirectly inhibiting vesicular uptake in the HEK-DAT/VMAT2 cells, counteracting the increased DAT-mediated uptake. This is likely an indirect modulation of VMAT2 because nuciferine did not directly affect VMAT2 uptake in the isolated vesicle assay. However, further experiments are necessary to determine the mechanism.

### 4.2 Behavioral characterization

Since the initial studies of Macko and colleagues, our ability to assess antipsychotic efficacy in animal models has improved. For example, deficits in sensorimotor gating observed in individuals diagnosed with schizophrenia are modeled in rodents using the pre-pulse inhibition (PPI) paradigm, in which the startle response to a stimulus is inhibited by a pulse of reduced magnitude preceding the acoustic startle stimulus. Deficits in PPI are observed in schizophrenia patients and can be rescued by antipsychotic medications.[58] This deficit in PPI can be reproduced in animal models via administration of phencyclidine (PCP), a compound that produces psychosis-like effects in humans, and the disrupted PPI can be rescued by antipsychotic compounds.[59, 60] Notably, the rescue of PCP-induced disruption of PPI has been a hallmark of preclinical antipsychotic drug discovery efforts as a predictive model of antipsychotic efficacy. [61]

We thus set out to determine whether the identified molecular profile would translate into antipsychotic drug-like actions as determined by several animal models commonly used to study antipsychotic pharmacology. As mentioned previously, atypical antipsychotics exhibit 5-HT_2A_ antagonist activity, and the potency with which antagonists attenuate the head-twitch response is highly correlated with the antagonist's affinity for 5-HT_2A_ receptors. [62, 63] The ability of nuciferine to block the hallucinogen DOI-induced head-twitch response is consistent with the *in vitro* measurements of 5-HT_2A_ antagonism and suggests activity *in vivo* via 5-HT_2A_ receptor blockade. Furthermore, the dissociative psychedelic PCP produces a psychomimetic state in humans and the inhibition of PCP-induced behavioral effects is used as an animal model for evaluating schizophrenia-like behaviors.[64, 65] Nuciferine blocked not only the PCP-induced hyperlocomotor activity in the open field, but it also rescued PCP-disrupted PPI without direct antagonism of the NMDA PCP binding site. Despite clozapine rescuing PPI in DAT-KO mice, nuciferine failed to normalize PPI in the DAT-KO genetic mouse model of hyperdopaminergia or in the pharmacological model using amphetamine (data not shown). At this time, the molecular basis of the distinctions between the differential responses in the hypoglutamateric and hyperdopaminergic models is obscure. To our knowledge, this is the first report of nuciferine’s efficacy in these animal models and our results indicate that this compound has greater efficacy in hypoglutamateric than in hyperdopaminergic animal models

The drug discrimination paradigm utilizes the interoceptive properties of drugs as a means to study their pharmacology. We used this paradigm to assess nuciferine’s pharmacology due to its unique ability to measure a systems-level pharmacological effect (as would be caused by a compound with high polypharmacology) at a whole organism level of analysis and due to its use in drug discovery efforts in the past.[66, 67] Nuciferine’s antagonism of the DOI-induced discriminative stimulus is consistent with its capacity to also antagonize DOI-induced head twitches and this result further validates the 5-HT_2A_ antagonism observed *in vitro*. Interestingly, nuciferine did not block the PCP-induced discriminative stimulus. It should be emphasized that other investigators have reported that antipsychotics do not antagonize the discriminative stimulus of PCP. [68] Finally, nuciferine fully substituted for clozapine at the highest dose tested (10.0 mg/kg), indicating that nuciferine produced an interoceptive state similar to that of clozapine. Previous studies in C57BL/6 mice have shown that 5-HT_2A_ serotonergic and α1-adrenoceptor antagonism mediate clozapine’s discriminative stimulus. [69] It should be noted that the 10.0 mg/kg dose of nuciferine that substituted for clozapine also produced significant rate suppression compared to vehicle control rates; however, previous studies have shown that the doses of antipsychotics that produce full substitution for clozapine are often accompanied by significant rate suppression (in both rats [70, 71] and mice[72, 73]).

### 4.3 Conclusion

In conclusion, we have comprehensively elucidated a complex pharmacological profile for nuciferine, one of the main alkaloids present in *Nelumbo nucifera*. The molecular profile of nuciferine was similar but not identical to the profiles of several approved antipsychotic drugs suggesting that nuciferine has atypical antipsychotic-like actions.

## References

[1] Emboden WA. Transcultural use of narcotic water lilies in ancient Egyptian and Maya drug ritual. Journal of ethnopharmacology. 1981;3(1):39–83. Epub 1981/01/01. . 7007741 10.1016/0378-8741(81)90013-1

[2] Zhang JY, Nawoschik S, Kowal D, Smith D, Spangler T, Ochalski R, et al Characterization of the 5-HT6 receptor coupled to Ca2+ signaling using an enabling chimeric G-protein. European journal of pharmacology. 2003;472(1–2):33–8. Epub 2003/07/16. doi: S0014299903018557 [pii]. . 12860470 10.1016/s0014-2999(03)01855-7

[3] Macko E, Douglas B, Weisbach JA, Waltz DT. Studies on the pharmacology of nuciferine and related aporphines. Archives internationales de pharmacodynamie et de therapie. 1972;197(2):261–73. Epub 1972/06/01. . 4402300

[4] Bhattacharya SK, Bose R, Ghosh P, Tripathi VJ, Ray AB, Dasgupta B. Psychopharmacological studies on (—)-nuciferine and its Hofmann degradation product atherosperminine. Psychopharmacology (Berl). 1978;59(1):29–33. Epub 1978/09/15. . 100809 10.1007/BF00428026

[5] Meltzer HY, Matsubara S, Lee JC. Classification of typical and atypical antipsychotic drugs on the basis of dopamine D-1, D-2 and serotonin2 pKi values. J Pharmacol Exp Ther. 1989;251(1):238–46. Epub 1989/10/01. . 2571717

[6] Roth BL, Sheffler DJ, Kroeze WK. Magic shotguns versus magic bullets: selectively non-selective drugs for mood disorders and schizophrenia. Nat Rev Drug Discov. 2004;3(4):353–9. Epub 2004/04/03. 10.1038/nrd1346 . 15060530

[7] Meltzer HY, Roth BL. Lorcaserin and pimavanserin: emerging selectivity of serotonin receptor subtype-targeted drugs. J Clin Invest. 2013;123(12):4986–91. Epub 2013/12/03. 10.1172/JCI7067870678 [pii]. 24292660 PMC3859385

[8] Yadav PN, Abbas AI, Farrell MS, Setola V, Sciaky N, Huang XP, et al The presynaptic component of the serotonergic system is required for clozapine's efficacy. Neuropsychopharmacology. 2011;36(3):638–51. Epub 2010/11/05. 10.1038/npp.2010.195 21048700 PMC3055689

[9] Besnard J, Ruda GF, Setola V, Abecassis K, Rodriguiz RM, Huang XP, et al Automated design of ligands to polypharmacological profiles. Nature. 2012;492(7428):215–20. Epub 2012/12/14. 10.1038/nature11691 . 23235874 PMC3653568

[10] Gu S, Zhu G, Wang Y, Li Q, Wu X, Zhang J, et al A sensitive liquid chromatography-tandem mass spectrometry method for pharmacokinetics and tissue distribution of nuciferine in rats. J Chromatogr B Analyt Technol Biomed Life Sci. 2014;961:20–8. 10.1016/j.jchromb.2014.04.038 . 24854711

[11] Mukherjee PK, Mukherjee D, Maji AK, Rai S, Heinrich M. The sacred lotus (Nelumbo nucifera)—phytochemical and therapeutic profile. J Pharm Pharmacol. 2009;61(4):407–22. Epub 2009/03/21. 10.1211/jpp/61.04.0001 . 19298686

[12] Gaulton A, Bellis LJ, Bento AP, Chambers J, Davies M, Hersey A, et al ChEMBL: a large-scale bioactivity database for drug discovery. Nucleic acids research. 2012;40(Database issue):D1100–7. Epub 2011/09/29. 10.1093/nar/gkr777 gkr777 [pii]. 21948594 PMC3245175

[13] Keiser MJ, Roth BL, Armbruster BN, Ernsberger P, Irwin JJ, Shoichet BK. Relating protein pharmacology by ligand chemistry. Nature biotechnology. 2007;25(2):197–206. Epub 2007/02/09. 10.1038/nbt1284 . 17287757

[14] Hert J, Keiser MJ, Irwin JJ, Oprea TI, Shoichet BK. Quantifying the relationships among drug classes. J Chem Inf Model. 2008;48(4):755–65. Epub 2008/03/14. 10.1021/ci8000259 18335977 PMC2722950

[15] Lin H, Sassano MF, Roth BL, Shoichet BK. A pharmacological organization of G protein-coupled receptors. Nat Methods. 2013;10(2):140–6. 10.1038/nmeth.2324 23291723 PMC3560304

[16] Bento AP, Gaulton A, Hersey A, Bellis LJ, Chambers J, Davies M, et al The ChEMBL bioactivity database: an update. Nucleic acids research. 2014;42(Database issue):D1083–90. 10.1093/nar/gkt1031 24214965 PMC3965067

[17] Liu H, Wang L, Lv M, Pei R, Li P, Pei Z, et al AlzPlatform: an Alzheimer's disease domain-specific chemogenomics knowledgebase for polypharmacology and target identification research. J Chem Inf Model. 2014;54(4):1050–60. Epub 2014/03/07. 10.1021/ci500004h 24597646 PMC4010297

[18] Roth BL, Baner K, Westkaemper R, Siebert D, Rice KC, Steinberg S, et al Salvinorin A: a potent naturally occurring nonnitrogenous kappa opioid selective agonist. Proc Natl Acad Sci U S A. 2002;99(18):11934–9. Epub 2002/08/23. 10.1073/pnas.182234399 182234399 [pii]. 12192085 PMC129372

[19] Shapiro DA, Renock S, Arrington E, Chiodo LA, Liu LX, Sibley DR, et al Aripiprazole, a novel atypical antipsychotic drug with a unique and robust pharmacology. Neuropsychopharmacology. 2003;28(8):1400–11. Epub 2003/06/05. 10.1038/sj.npp.1300203 1300203 [pii]. . 12784105

[20] Keiser MJ, Setola V, Irwin JJ, Laggner C, Abbas AI, Hufeisen SJ, et al Predicting new molecular targets for known drugs. Nature. 2009;462(7270):175–81. Epub 2009/11/03. 10.1038/nature08506 nature08506 [pii]. 19881490 PMC2784146

[21] Feng BY, Simeonov A, Jadhav A, Babaoglu K, Inglese J, Shoichet BK, et al A high-throughput screen for aggregation-based inhibition in a large compound library. J Med Chem. 2007;50(10):2385–90. Epub 2007/04/24. 10.1021/jm061317y . 17447748

[22] Abbas AI, Yadav PN, Yao WD, Arbuckle MI, Grant SG, Caron MG, et al PSD-95 is essential for hallucinogen and atypical antipsychotic drug actions at serotonin receptors. J Neurosci. 2009;29(22):7124–36. Epub 2009/06/06. doi: 29/22/7124 [pii] 10.1523/JNEUROSCI.1090-09.2009 . 19494135 PMC2836830

[23] Corne SJ, Pickering RW, Warner BT. A method for assessing the effects of drugs on the central actions of 5-hydroxytryptamine. British journal of pharmacology and chemotherapy. 1963;20:106–20. 14023050 10.1111/j.1476-5381.1963.tb01302.x PMC1703746

[24] Fantegrossi WE, Kiessel CL, Leach PT, Van Martin C, Karabenick RL, Chen X, et al Nantenine: an antagonist of the behavioral and physiological effects of MDMA in mice. Psychopharmacology (Berl). 2004;173(3–4):270–7. 10.1007/s00213-003-1741-2 . 14740148

[25] Fantegrossi WE, Murai N, Mathuna BO, Pizarro N, de la Torre R. Discriminative stimulus effects of 3,4-methylenedioxymethamphetamine and its enantiomers in mice: pharmacokinetic considerations. J Pharmacol Exp Ther. 2009;329(3):1006–15. Epub 2009/03/12. 10.1124/jpet.109.150573 jpet.109.150573 [pii]. 19276400 PMC2683777

[26] Fantegrossi WE, Reissig CJ, Katz EB, Yarosh HL, Rice KC, Winter JC. Hallucinogen-like effects of N,N-dipropyltryptamine (DPT): possible mediation by serotonin 5-HT1A and 5-HT2A receptors in rodents. Pharmacol Biochem Behav. 2008;88(3):358–65. Epub 2007/10/02. doi: S0091-3057(07)00289-4 [pii] 10.1016/j.pbb.2007.09.007 17905422 PMC2322878

[27] Wiebelhaus JM, Vunck SA, Meltzer HY, Porter JH. Discriminative stimulus properties of N-desmethylclozapine, the major active metabolite of the atypical antipsychotic clozapine, in C57BL/6 mice. Behavioural pharmacology. 2012;23(3):262–70. Epub 2012/05/02. 10.1097/FBP.0b013e3283534332 00008877-201206000-00005 [pii]. . 22547022

[28] Extance K, Goudie AJ. Inter-animal olfactory cues in operant drug discrimination procedures in rats. Psychopharmacology (Berl). 1981;73(4):363–71. Epub 1981/01/01. . 6789359 10.1007/BF00426467

[29] Krobert KA, Levy FO. The human 5-HT7 serotonin receptor splice variants: constitutive activity and inverse agonist effects. British journal of pharmacology. 2002;135(6):1563–71. Epub 2002/03/22. 10.1038/sj.bjp.0704588 11906971 PMC1573253

[30] Creese I, Burt DR, Snyder SH. Dopamine receptor binding predicts clinical and pharmacological potencies of antischizophrenic drugs. Science. 1976;192(4238):481–3. Epub 1976/04/30. . 3854 10.1126/science.3854

[31] Burris KD, Molski TF, Xu C, Ryan E, Tottori K, Kikuchi T, et al Aripiprazole, a novel antipsychotic, is a high-affinity partial agonist at human dopamine D2 receptors. J Pharmacol Exp Ther. 2002;302(1):381–9. Epub 2002/06/18. . 12065741 10.1124/jpet.102.033175

[32] Kenakin T. Drugs and receptors. An overview of the current state of knowledge. Drugs. 1990;40(5):666–87. Epub 1990/11/01. . 2292230 10.2165/00003495-199040050-00003

[33] Thomas DR, Gittins SA, Collin LL, Middlemiss DN, Riley G, Hagan J, et al Functional characterisation of the human cloned 5-HT7 receptor (long form); antagonist profile of SB-258719. British journal of pharmacology. 1998;124(6):1300–6. Epub 1998/08/28. 10.1038/sj.bjp.0701946 9720804 PMC1565501

[34] Roth BL, Craigo SC, Choudhary MS, Uluer A, Monsma FJ Jr., Shen Y, et al Binding of typical and atypical antipsychotic agents to 5-hydroxytryptamine-6 and 5-hydroxytryptamine-7 receptors. J Pharmacol Exp Ther. 1994;268(3):1403–10. Epub 1994/03/01. . 7908055

[35] McGovern SL, Caselli E, Grigorieff N, Shoichet BK. A common mechanism underlying promiscuous inhibitors from virtual and high-throughput screening. J Med Chem. 2002;45(8):1712–22. Epub 2002/04/05. doi: jm010533y [pii]. . 11931626 10.1021/jm010533y

[36] Ferreira RS, Simeonov A, Jadhav A, Eidam O, Mott BT, Keiser MJ, et al Complementarity between a docking and a high-throughput screen in discovering new cruzain inhibitors. J Med Chem. 2010;53(13):4891–905. Epub 2010/06/15. 10.1021/jm100488w 20540517 PMC2895358

[37] Sassano MF, Doak AK, Roth BL, Shoichet BK. Colloidal aggregation causes inhibition of G protein-coupled receptors. J Med Chem. 2013;56(6):2406–14. Epub 2013/02/27. 10.1021/jm301749y 23437772 PMC3613083

[38] Duan D, Doak AK, Nedyalkova L, Shoichet BK. Colloidal aggregation and the in vitro activity of traditional Chinese medicines. ACS chemical biology. 2015;10(4):978–88. 10.1021/cb5009487 . 25606714 PMC4646422

[39] Bernstein AI, Stout KA, Miller GW. A fluorescent-based assay for live cell, spatially resolved assessment of vesicular monoamine transporter 2-mediated neurotransmitter transport. Journal of neuroscience methods. 2012;209(2):357–66. Epub 2012/06/16. 10.1016/j.jneumeth.2012.06.002 22698664 PMC3429701

[40] Mahe C, Loetscher E, Feuerbach D, Muller W, Seiler MP, Schoeffter P. Differential inverse agonist efficacies of SB-258719, SB-258741 and SB-269970 at human recombinant serotonin 5-HT7 receptors. European journal of pharmacology. 2004;495(2–3):97–102. Epub 2004/07/14. 10.1016/j.ejphar.2004.05.033 S0014299904005527 [pii]. . 15249157

[41] Ishibashi T, Horisawa T, Tokuda K, Ishiyama T, Ogasa M, Tagashira R, et al Pharmacological profile of lurasidone, a novel antipsychotic agent with potent 5-hydroxytryptamine 7 (5-HT7) and 5-HT1A receptor activity. J Pharmacol Exp Ther. 2010;334(1):171–81. Epub 2010/04/21. 10.1124/jpet.110.167346 . 20404009

[42] Newman-Tancredi A, Kleven MS. Comparative pharmacology of antipsychotics possessing combined dopamine D2 and serotonin 5-HT1A receptor properties. Psychopharmacology (Berl). 2011;216(4):451–73. Epub 2011/03/12. 10.1007/s00213-011-2247-y . 21394633

[43] Audinot V, Newman-Tancredi A, Cussac D, Millan MJ. Inverse agonist properties of antipsychotic agents at cloned, human (h) serotonin (5-HT)(1B) and h5-HT(1D) receptors. Neuropsychopharmacology. 2001;25(3):410–22. Epub 2001/08/28. 10.1016/S0893-133X(01)00237-8 . 11522469

[44] Masri B, Salahpour A, Didriksen M, Ghisi V, Beaulieu JM, Gainetdinov RR, et al Antagonism of dopamine D2 receptor/beta-arrestin 2 interaction is a common property of clinically effective antipsychotics. Proc Natl Acad Sci U S A. 2008;105(36):13656–61. Epub 2008/09/05. 10.1073/pnas.0803522105 18768802 PMC2533245

[45] Sokoloff P, Diaz J, Le Foll B, Guillin O, Leriche L, Bezard E, et al The dopamine D3 receptor: a therapeutic target for the treatment of neuropsychiatric disorders. CNS & neurological disorders drug targets. 2006;5(1):25–43. Epub 2006/04/15. . 16613552 10.2174/187152706784111551

[46] Shahid M, Walker GB, Zorn SH, Wong EH. Asenapine: a novel psychopharmacologic agent with a unique human receptor signature. Journal of psychopharmacology. 2009;23(1):65–73. Epub 2008/03/01. 10.1177/0269881107082944 . 18308814

[47] Shoichet BK. Screening in a spirit haunted world. Drug discovery today. 2006;11(13–14):607–15. Epub 2006/06/24. 10.1016/j.drudis.2006.05.014 16793529 PMC1524586

[48] Owen SC, Doak AK, Wassam P, Shoichet MS, Shoichet BK. Colloidal aggregation affects the efficacy of anticancer drugs in cell culture. ACS chemical biology. 2012;7(8):1429–35. Epub 2012/05/26. 10.1021/cb300189b 22625864 PMC3423826

[49] Terstappen GC, Pula G, Carignani C, Chen MX, Roncarati R. Pharmacological characterisation of the human small conductance calcium-activated potassium channel hSK3 reveals sensitivity to tricyclic antidepressants and antipsychotic phenothiazines. Neuropharmacology. 2001;40(6):772–83. Epub 2001/05/23. . 11369031 10.1016/s0028-3908(01)00007-7

[50] Zheng G, Dwoskin LP, Crooks PA. Vesicular monoamine transporter 2: role as a novel target for drug development. The AAPS journal. 2006;8(4):E682–92. Epub 2007/01/20. 10.1208/aapsj080478 17233532 PMC2751365

[51] Kinross-Wright V. Chlorpromazine and reserpine in the treatment of psychoses. Annals of the New York Academy of Sciences. 1955;61(1):174–82. Epub 1955/04/15. . 14377285 10.1111/j.1749-6632.1955.tb42464.x

[52] De Hert M, Wampers M, van Winkel R, Peuskens J. Anticholinergic use in hospitalised schizophrenic patients in Belgium. Psychiatry research. 2007;152(2–3):165–72. Epub 2007/04/21. 10.1016/j.psychres.2006.07.012 . 17445906

[53] Peluso MJ, Lewis SW, Barnes TR, Jones PB. Extrapyramidal motor side-effects of first- and second-generation antipsychotic drugs. The British journal of psychiatry: the journal of mental science. 2012;200(5):387–92. Epub 2012/03/24. 10.1192/bjp.bp.111.101485 . 22442101

[54] Desmarais JE, Beauclair L, Margolese HC. Anticholinergics in the era of atypical antipsychotics: short-term or long-term treatment? Journal of psychopharmacology. 2012;26(9):1167–74. Epub 2012/06/02. 10.1177/0269881112447988 . 22651987

[55] Munusamy V, Yap BK, Buckle MJ, Doughty SW, Chung LY. Structure-Based Identification of Aporphines with Selective 5-HT(2A) Receptor-Binding Activity. Chemical biology & drug design. 2012 Epub 2012/10/09. 10.1111/cbdd.12069 . 23039820

[56] Tadori Y, Miwa T, Tottori K, Burris KD, Stark A, Mori T, et al Aripiprazole's low intrinsic activities at human dopamine D2L and D2S receptors render it a unique antipsychotic. European journal of pharmacology. 2005;515(1–3):10–9. Epub 2005/05/17. doi: S0014-2999(05)00402-4 [pii] 10.1016/j.ejphar.2005.02.051 . 15894311

[57] Etievant A, Betry C, Arnt J, Haddjeri N. Bifeprunox and aripiprazole suppress in vivo VTA dopaminergic neuronal activity via D2 and not D3 dopamine autoreceptor activation. Neurosci Lett. 2009;460(1):82–6. Epub 2009/05/20. 10.1016/j.neulet.2009.05.035 S0304-3940(09)00659-4 [pii]. . 19450663

[58] Braff DL, Geyer MA. Sensorimotor gating and schizophrenia. Human and animal model studies. Arch Gen Psychiatry. 1990;47(2):181–8. Epub 1990/02/01. . 2405807 10.1001/archpsyc.1990.01810140081011

[59] Geyer MA, Krebs-Thomson K, Braff DL, Swerdlow NR. Pharmacological studies of prepulse inhibition models of sensorimotor gating deficits in schizophrenia: a decade in review. Psychopharmacology (Berl). 2001;156(2–3):117–54. Epub 2001/09/11. . 11549216 10.1007/s002130100811

[60] Bakshi VP, Swerdlow NR, Geyer MA. Clozapine antagonizes phencyclidine-induced deficits in sensorimotor gating of the startle response. J Pharmacol Exp Ther. 1994;271(2):787–94. Epub 1994/11/01. . 7965797

[61] Porsolt RD, Moser PC, Castagne V. Behavioral indices in antipsychotic drug discovery. J Pharmacol Exp Ther. 2010;333(3):632–8. Epub 2010/03/05. 10.1124/jpet.110.166710jpet.110.166710 [pii]. . 20200119

[62] Peroutka SJ, Lebovitz RM, Snyder SH. Two distinct central serotonin receptors with different physiological functions. Science. 1981;212(4496):827–9. . 7221567 10.1126/science.7221567

[63] Ortmann R, Bischoff S, Radeke E, Buech O, Delini-Stula A. Correlations between different measures of antiserotonin activity of drugs. Study with neuroleptics and serotonin receptor blockers. Naunyn Schmiedebergs Arch Pharmacol. 1982;321(4):265–70. . 6132341 10.1007/BF00498511

[64] Gonzalez-Maeso J, Sealfon SC. Psychedelics and schizophrenia. Trends Neurosci. 2009;32(4):225–32. Epub 2009/03/10. 10.1016/j.tins.2008.12.005 . 19269047

[65] Javitt DC, Zukin SR. Recent advances in the phencyclidine model of schizophrenia. Am J Psychiatry. 1991;148(10):1301–8. Epub 1991/10/01. . 1654746 10.1176/ajp.148.10.1301

[66] Colpaert FC. Drug discrimination in neurobiology. Pharmacol Biochem Behav. 1999;64(2):337–45. Epub 1999/10/09. . 10515310 10.1016/s0091-3057(99)00047-7

[67] Colpaert FC. Discovering risperidone: the LSD model of psychopathology. Nat Rev Drug Discov. 2003;2(4):315–20. Epub 2003/04/02. 10.1038/nrd1062 . 12669030

[68] Compton AD, Slemmer JE, Drew MR, Hyman JM, Golden KM, Balster RL, et al Combinations of clozapine and phencyclidine: effects on drug discrimination and behavioral inhibition in rats. Neuropharmacology. 2001;40(2):289–97. Epub 2000/12/15. . 11114408 10.1016/s0028-3908(00)00126-x

[69] Philibin SD, Walentiny DM, Vunck SA, Prus AJ, Meltzer HY, Porter JH. Further characterization of the discriminative stimulus properties of the atypical antipsychotic drug clozapine in C57BL/6 mice: role of 5-HT(2A) serotonergic and alpha (1) adrenergic antagonism. Psychopharmacology (Berl). 2009;203(2):303–15. Epub 2008/11/08. 10.1007/s00213-008-1385-3 . 18989659

[70] Prus AJ, Philibin SD, Pehrson AL, Porter JH. Discriminative stimulus properties of the atypical antipsychotic drug clozapine in rats trained to discriminate 1.25 mg/kg clozapine vs. 5.0 mg/kg clozapine vs. vehicle. Behavioural pharmacology. 2006;17(2):185–94. Epub 2006/02/24. 10.1097/01.fbp.0000197457.70774.91 . 16495726

[71] Porter JH, Prus AJ, Vann RE, Varvel SA. Discriminative stimulus properties of the atypical antipsychotic clozapine and the typical antipsychotic chlorpromazine in a three-choice drug discrimination procedure in rats. Psychopharmacology (Berl). 2005;178(1):67–77. Epub 2004/08/19. 10.1007/s00213-004-1985-5 . 15316715

[72] Philibin SD, Prus AJ, Pehrson AL, Porter JH. Serotonin receptor mechanisms mediate the discriminative stimulus properties of the atypical antipsychotic clozapine in C57BL/6 mice. Psychopharmacology (Berl). 2005;180(1):49–56. Epub 2005/02/08. 10.1007/s00213-005-2147-0 15696329 PMC1351031

[73] Porter JH, Walentiny DM, Philibin SD, Vunck SA, Crabbe JC. A comparison of the discriminative stimulus properties of the atypical antipsychotic drug clozapine in DBA/2 and C57BL/6 inbred mice. Behavioural pharmacology. 2008;19(5–6):530–42. Epub 2008/08/12. 10.1097/FBP.0b013e32830cd84e . 18690107

[74] Lindsley CW. The top prescription drugs of 2012 globally: biologics dominate, but small molecule CNS drugs hold on to top spots. ACS Chem Neurosci. 2013;4(6):905–7. Epub 2013/09/13. 10.1021/cn400107y 24024784 PMC3689196

